# Tolerance measured at one developmental stage mispredicts climate resilience in an animal with a complex life cycle

**DOI:** 10.1371/journal.pbio.3004079

**Published:** 2026-10-26

**Authors:** Hao Song, Tingyu Shi, Meijie Yang, Xiaoyan Liu, Zhuoqing Li, Pu Shi, Yu Wu, Bassem Allam, Andrew Jeffs, Bradley Skelton, Tao Zhang

**Affiliations:** 1 Laboratory of Marine Ecology and Environmental Sciences, Institute of Oceanology, Chinese Academy of Sciences, Qingdao, China; 2 School of Marine Sciences, Ningbo University, Ningbo, China; 3 Laboratory for Marine Ecology and Environmental Science, Qingdao Marine Science and Technology Center, Qingdao, China; 4 State Key Laboratory of Breeding Biotechnology and Sustainable Aquaculture, Institute of Oceanology, Chinese Academy of Sciences, Qingdao, China; 5 University of Chinese Academy of Sciences, Beijing, China; 6 Marine Animal Disease Laboratory, School of Marine and Atmospheric Sciences, Stony Brook University, Stony Brook, New York, United States of America; 7 Institute of Marine Science, University of Auckland, Auckland, New Zealand; 8 Lianyungang Zhongke Meibei Marine Technology Co., Ltd, Lianyungang, China; University of Washington Seattle Campus: University of Washington, UNITED STATES OF AMERICA

## Abstract

Forecasts of whether marine species can evolve fast enough to survive climate change rely largely on single-stage tolerance assays, tacitly assuming that tolerance measured at one developmental stage reflects adaptive capacity across the organism’s life cycle. We test this assumption in a common marine bivalve (*Mercenaria mercenaria*) and find that it fails in sign, not degree: larval and post-metamorphic tolerance to end-century ocean acidification are strongly negatively correlated (*r* = −0.53, 95% CI [−0.69, −0.33]) across family-level, pedigree-conditioned and animal model estimates, with pervasive rank reversals among the top performers of each stage. A multivariate analysis of the among-family covariance matrix G shows why: under acidification, 57% of the among-family variance collapses onto a single dominant axis that loads positively on every larval trait and negatively on every post-metamorphic trait, whereas under ambient conditions the same families show no cross-stage covariance at all. Acidification therefore does not merely depress performance—it reorganizes the genetic architecture into a cross-stage conflict. Under ramping RCP 8.5 forcing this conflict geometry dictates the direction—not merely the magnitude—of adaptation: only balanced multi-stage selection preserves family diversity, whereas single-stage selection systematically erodes the opposite axis. In complex life cycles, resilience is a geometric property of ontogeny that forecasts cannot compress into a single-stage without inverting the sign of the answer.

## Abstract 2

Forecasts of whether marine species can survive climate change rely largely on tolerance assays at one developmental stage. This study of the tolerance of an animal with a complex life cycle to end-century ocean acidification reveals cross-stage conflicts that impact the direction and magintude of adaptation.

## Introduction

Anthropogenic CO_2_ has reshaped surface-ocean carbonate chemistry at planetary scale, and meta-analyses have long established that it depresses calcification, development and recruitment across a wide range of marine taxa [1–4]. For marine animals with complex life cycles, however, the key uncertainty is not simply whether ocean acidification (OA) harms larvae, juveniles, or adults, but whether tolerance measured at one developmental stage predicts adaptive capacity across the organism’s life cycle. This distinction matters because roughly 80% of marine invertebrates carry a bipartite or multi-stage life history [5–8], yet the dominant experimental paradigm in ocean acidification biology measures tolerance at one developmental stage—most often the larval phase—and extrapolates from it [9–15]. This workflow is understandable: larval assays are tractable, scalable, and often capture the stage at which mortality is most severe. But larvae and post-metamorphic animals face different physiological problems. Larvae must build a first shell and complete development within a narrow temporal window, whereas juveniles and adults must sustain shell maintenance, acid–base regulation, growth and reproduction over much longer time scales. A family that performs well at one stage therefore need not perform well at another. The biological question is whether single-stage tolerance is an adequate proxy for life-cycle tolerance, or whether metamorphosis changes the axis on which acidification tolerance is expressed.

In evolutionary terms, this is a problem of covariance. The direction in which selection can move a complex-life-history population is set not only by the strength of selection within each stage, but also by the cross-stage covariance of trait-mediated fitness—the off-diagonal structure of the multivariate G-matrix [16–20]. Standing genetic variation can be abundant at each stage yet still fail to generate favorable life-cycle adaptation if the selection vectors acting before and after metamorphosis are not aligned in the same genetic coordinates [19,20]. Because larval and post-metamorphic stages differ in size, exposure, physiology and fitness bookkeeping, the same allele, genotype, or family can occupy different positions on different adaptive surfaces across ontogeny [5–10,12,13,21,22]. In a Fisher geometric framework [23–25], movement toward the optimum on one axis can move performance away from the optimum on another; the same logic applies across life stages whenever stage-specific selection vectors are noncollinear [26–29].

These geometric considerations are not abstract. They also explain why, despite two decades of OA experiments on bivalves, sea urchins and other calcifiers, the cross-stage structure of tolerance has remained poorly characterized [9–15]. Larval and adult assays typically operate on different biological material: larval tolerance is screened at the vial or well-plate level on mass-spawned cohorts, whereas adult performance is usually measured on independently sourced juveniles or adults, and the two datasets are rarely connected by a shared genetic scaffold [11–13]. This matters because a cross-stage trade-off can only be seen if the same biological units—the same families, genotypes, or lineages—are measured before and after metamorphosis. If larval and adult performance are scored on different animals, even a strong trade-off leaves no trace in the data [20,29,30]. A systematic map of the marine complex-life-history literature confirms how rarely this condition is met: of 3,315 experiments, 67% measured a single-stage and only 1.7% tracked the same individuals across stages [31]. The result is an experimental literature that can document severe impacts at each stage while remaining silent on whether the same biological lineages—families, genotypes, alleles—that thrive as larvae will also thrive as adults. If the cross-stage covariance is close to zero, this silence is harmless. If it is negative, the silence is consequential: the field can then accumulate confident forecasts pointing in the wrong direction.

Three possible scenarios emerge (Fig 1). A whole-organism integration model predicts positive cross-stage covariance because a largely shared tolerance architecture benefits both stages (Fig 1a). An ontogenetic decoupling model predicts near-zero covariance because metamorphosis genetically buffers stages from one another [7,8] (Fig 1b). A third possibility—less frequently tested but more consequential—is stage-partitioned conflict, in which tolerance gained at one stage is gained at the expense of tolerance at another (Fig 1c). Under this third model, OA does not simply depress performance at each stage in parallel: it reshapes the fitness landscape so that the same larval-acting selection produces categorically different adaptive trajectories depending on the sign of the cross-stage covariance [19,20,26–29] (Fig 1d–1f). Stage-specific selection on the most vulnerable phase can then become a genetic bottleneck, concentrating the breeding pool around families that traverse one filter while eroding variance along another life-stage axis [19,20,30]. Which of these three scenarios describes OA tolerance is therefore a first-order question for climate-adaptation forecasting, not a technical footnote. Population-genetic theory for complex life histories makes the third of them the expected outcome rather than an exotic one: extensions of Fisher’s geometric model show that pleiotropic trade-offs between life-cycle stages arise readily from both divergent selection and mutation, and that conflict between stages escalates as adaptation proceeds [32,33].

**Fig 1:**
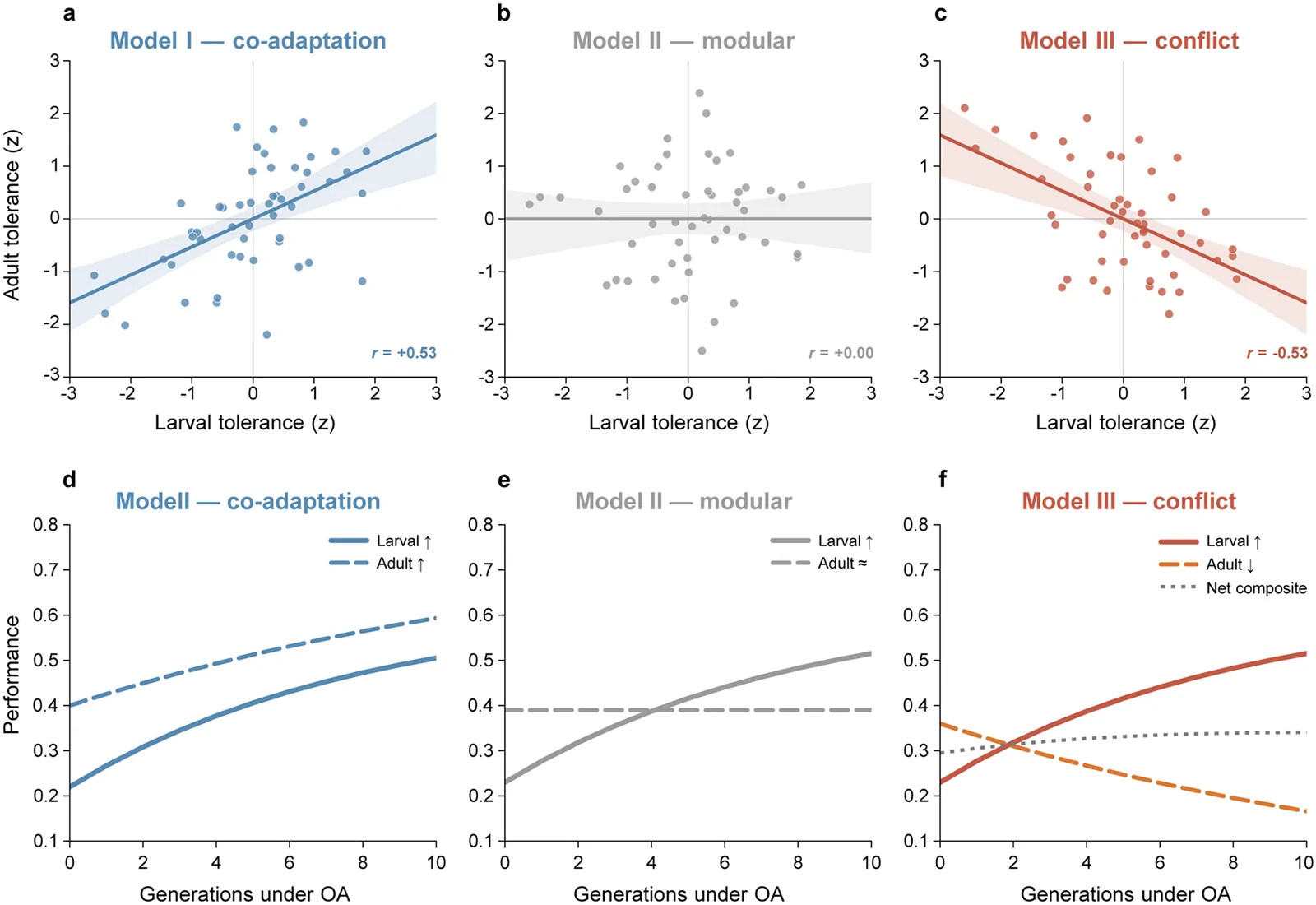
Three competing models show that climate tolerance can integrate, decouple or reverse across metamorphosis. This figure is a conceptual schematic, not data: the points and lines are generated from a simulation in which 51 hypothetical families are drawn from bivariate normal distributions with imposed cross-stage correlations of +0.53, 0.00 and −0.53; the trajectories in d–f are schematic curves illustrating the qualitative expectation under each architecture. It illustrates the three hypotheses the experiment is designed to distinguish and the trajectory signatures each would produce; the corresponding empirical results are shown in Figs 2 and 6. **a–c,** Three alternative cross-stage architectures for family-level larval and post-metamorphic OA tolerance: positive covariance (co-adaptation), zero covariance (modularity) and negative covariance (conflict). Lines show the simulated relationships; shaded bands indicate 95% bootstrap intervals on the simulated draws. **d–f,** Expected trajectory signatures under uniform larval-acting selection. Positive covariance improves both stages, modularity improves the selected stage without changing the other, and conflict improves larval performance while eroding post-metamorphic performance, producing a lower net life-cycle composite. The numerical data underlying this figure are provided in https://doi.org/10.6084/m9.figshare.34030317.

The prediction is deliberately strong: all three hypotheses are mathematically compatible with OA-depressed performance at both stages, and distinguishing among them requires matched-stage phenotypes on the same genetic units, not separate single-stage datasets compared post hoc. Only within a shared pedigree can cross-stage covariance be identified independently of differential survival, differential sampling across stages, or differential environmental exposure. This identifiability requirement has been noted in the quantitative-genetic literature on multivariate selection [20,29,30] but rarely implemented in OA biology, where hatchery-level logistics tend to force stage-separate experiments.

Here we provide such a test. We bred 51 families of the hard clam *M. mercenaria* in a partial half-sib pedigree (12 sires × 6 dams) and measured the same families both before and after metamorphosis, under present-day and end-century acidification. This lets us ask three questions that single-stage designs cannot: do the families that tolerate acidification best as larvae also tolerate it best as juveniles and adults; is any cross-stage relationship a property of the multivariate genetic architecture rather than of one summary score; and, if the relationship is negative, what does that imply for populations evolving under sustained acidification? We answer the first with matched stage-specific tolerance scores, the second with the among-family covariance matrix G and its principal axes, and the third with a transparent 20-generation model in which families are re-weighted each generation according to their relative fitness. Nor is this merely a concern for bivalves. Whenever a complex-life-history species has been treated as a single adaptive unit for the purposes of climate-tolerance forecasting—corals, sea urchins, crustaceans, many polychaetes and most commercially exploited mollusks [8,13,21,34,35]—the same identifiability problem applies. Our question is therefore not whether OA harms one stage more than another, but whether the fitness axis itself can bend between stages, and what happens to adaptation when it does.

## Results

To move from the three conceptual models to an empirical test, we first anchored every stage-specific measurement to the same genetic units. Each operational family was propagated through fully replicated ambient (pH_T_ 8.05 ± 0.02, Ω_arag_ 2.18) and acidified (pH_T_ 7.69 ± 0.02, Ω_arag_ 0.98) seawater systems with stable carbonate chemistry (S1 and S2 Figs; S1 Table). Larval traits (survival, shell area, thickness, hardness and days to metamorphosis) were measured at vial level within a block → tank → vial hierarchy. Post-metamorphic traits (shell growth, dissolution and MO_2_, the mass-specific rate of oxygen uptake) were measured on individual juveniles after a 90-day OA nursery, and gonadal index was measured on the same animals after two further years in a common ambient garden. The two exposures were independent—no animal was acidified at both stages—so the larval and post-metamorphic measurements are connected only by shared family membership (Materials and methods, §4). Gonadal index is therefore a delayed consequence of the animal’s own earlier post-metamorphic exposure, not of anything that happened to it as a larva. All analyses retained the nested random-effect structure of the design, and the partial half-sib pedigree provided the numerator-relationship scaffold for the bivariate pedigree animal model (S2 Table). We therefore begin with the question that decides between the three models—whether family rankings are preserved or inverted across metamorphosis—and then turn to the stage-specific filters, and to the multivariate genetic architecture, that explain the inversion.

### Metamorphosis reverses family rankings for climate tolerance

We first tested the strongest prediction of the conflict model: the families that look climate-resilient before metamorphosis should not be the same families that look resilient afterward. For each family, we calculated stage-specific composite tolerance as weighted geometric means of OA/ambient trait-retention ratios, so that tolerance reflects stage-wide performance rather than a single endpoint (Materials and methods, Eqs. 2 and 3; S3 Table). The cross-stage answer was strongly negative: larval and post-metamorphic tolerance were inversely associated across families (Pearson *r* = −0.530, 95% CI [−0.691, −0.332], *P* = 6.2 × 10^−5^; Spearman *ρ* = −0.548, *P* = 3.1 × 10^−5^; Fig 2a). The reversal was visible at the level that matters for selection and screening. Of the 13 families in the top larval quartile, 10 fell below the adult median; of the 13 families in the top adult quartile, 11 fell below the larval median; no family occupied the top quartile at both stages (Fig 2b, 2c). Thus the first empirical result is not simply that OA affects two stages differently, but that metamorphosis changes which genetic families look resilient.

**Fig 2:**
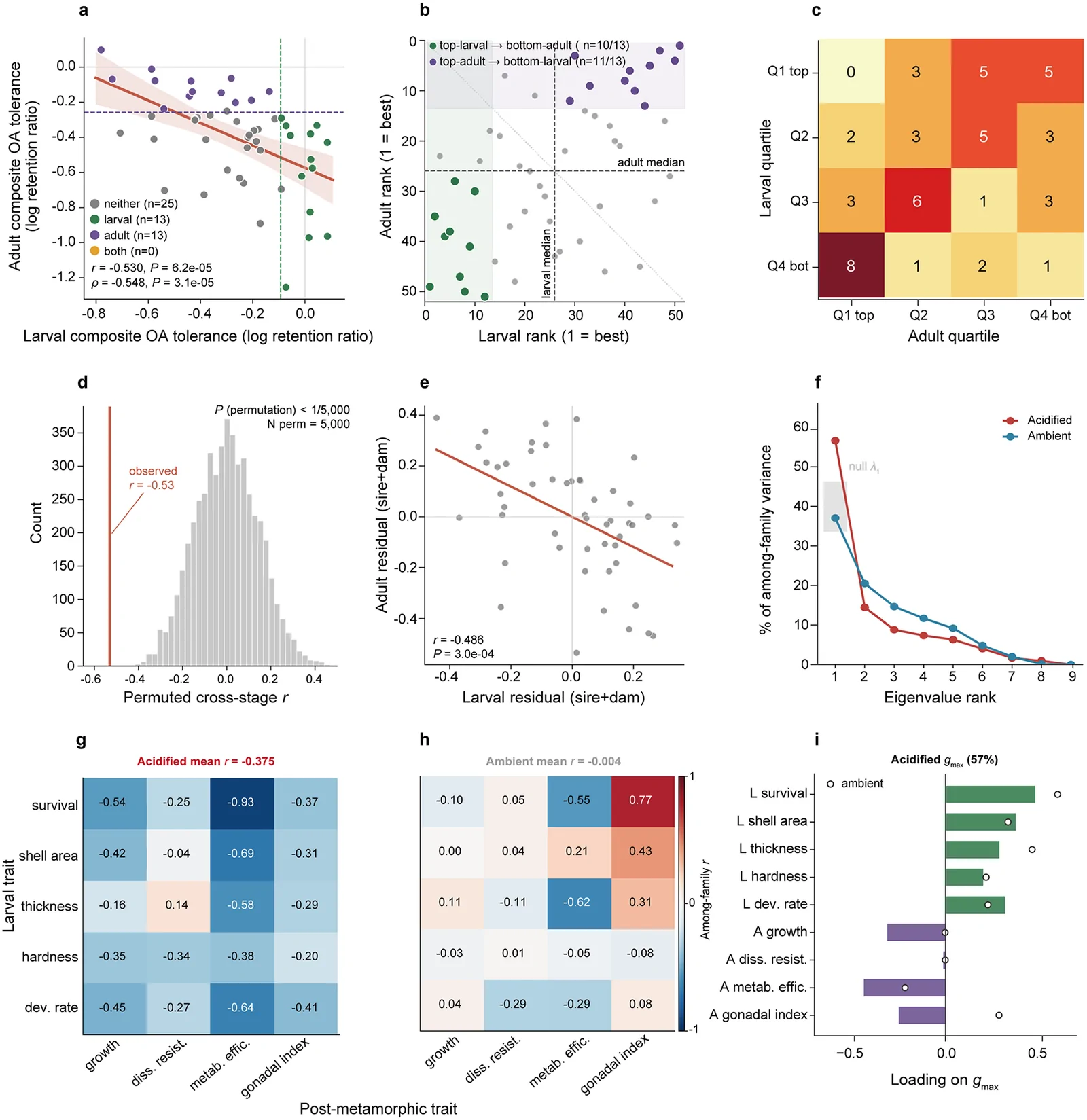
Metamorphosis reverses family rankings, and acidification collapses the multivariate architecture onto that conflict axis. **a,** Family-level larval and post-metamorphic composite OA tolerance for the 51 families, colored by whether a family falls in the top quartile at the larval stage, the post-metamorphic stage, both or neither. Both axes are log retention ratios (Eqs. 2, 3), so 0 marks a family whose performance under acidification equals its performance under ambient conditions and negative values denote a net loss; gray solid lines mark these zeroes and colored dashed lines mark the upper-quartile cut-offs used throughout. The negative cross-stage association shows that families ranked as larval winners tend to become post-metamorphic losers. **b,** Rank–rank plot. Dashed black lines mark the median rank at each stage; green and purple fills identify the top-quartile families of each stage, and red rings identify the 10 of 13 top-larval families that fall below the post-metamorphic median and the 11 of 13 top-post-metamorphic families that fall below the larval median. **c,** Quartile-overlap matrix; no family occupies the top quartile at both stages. **d,** Permutation null from 5,000 shuffles of family identity, placing the observed correlation (solid red line) in the extreme negative tail. **e,** Sire- and dam-conditioned residual correlation; the gray lines mark zero on each axis, i.e., a family performing exactly at its sire + dam expectation. The leave-one-sire and leave-one-dam diagnostic is shown in S3d Fig. **f,** Eigenvalue spectrum of the nine-trait among-family covariance matrix G in each environment, as a percentage of total among-family variance; the gray bar shows the 95% range of the leading eigenvalue under a design-based null in which cross-stage covariance is zero, but all other features of the design are preserved. **g,h,** Among-family correlations between each larval trait (rows) and each post-metamorphic trait (columns), estimated within the acidified (*g*) and the ambient (*h*) environment from the same 51 families. ***i,*** Trait loadings on the leading eigenvector $g_{\text{max}}$ under acidification (bars; green larval, purple post-metamorphic) with the ambient $g_{\text{max}}$ overlaid as open circles. Under acidification, every larval trait loads positively and every post-metamorphic trait negatively; under ambient conditions the post-metamorphic loadings show no consistent sign. The numerical data underlying this figure are provided at https://doi.org/10.6084/m9.figshare.34030317.

### The reversal is robust to pedigree structure and tolerance definitions

We next asked whether the inversion was a structural property of the pedigree rather than a consequence of one scoring rule or a few influential families. A 5,000-shuffle permutation null placed the observed cross-stage correlation in the extreme negative tail (*P* < 1/5,000; Fig 2d; S4 Table). Removing sire and dam structure left a negative residual association (*r* = −0.486, *P* = 3.0 × 10^−4^; Fig 2e), and leave-one-sire/dam analyses never changed the sign (S3d Fig). The result also survived five alternative tolerance definitions, including equal-weight geometric means, arithmetic means of ratio terms, and adult composites omitting gonadal index or MO_2_ (S3 Fig; S5 Table). Quantitative-genetic summaries pointed in the same direction: collapsing to the 12 shared sires gave *r* = −0.66 (95% CI [−0.90, −0.14], *P* = 0.019), dam-level summaries remained negative, and a bivariate pedigree animal model supported a negative cross-stage genetic correlation (S4 Fig; S6 Table). The sign is therefore not a family-outlier effect, not a composite-weight artifact and not created by ignoring the partial pedigree.

### Ocean acidification imposes different filters before and after metamorphosis

Having established the inversion, we then asked how acidification imposes within-stage filters that could generate it. In larvae, OA reduced vial-level survival from 0.573 ± 0.004 s.e. to 0.432 ± 0.007 s.e. (*β* = −0.587 ± 0.028 s.e.; Wald *χ*^2^ = 439.56, *P* < 0.001; S7 Table; Fig 3a, 3b). Shell area, shell thickness and shell hardness declined, whereas days to metamorphosis increased by 1.89 d (all *P* < 0.001; S8–S11 Tables; Fig 3c–3f). Importantly, acidification did not erase selectable family variance: family-level intraclass correlations (ICCs) were 0.187 for survival, 0.337 for shell area, 0.314 for thickness, 0.289 for hardness and 0.258 for metamorphosis (Fig 3g, 3h). Using the Lande–Arnold/Arnold–Wade selection-analysis framework [17,36], we then found that OA steepened directional selection toward larger first-shell area and faster metamorphosis (Fig 4a,4b): the standardized selection differential for shell area rose from +0.17 under ambient conditions to +0.28 under OA, and that for days to metamorphosis from −0.10 to −0.21 (treatment × trait interaction *P* < 10^−5^ for both; S12 Table), narrowing larval fitness around a high-calcification, rapid-development combination (Fig 4c,4d), with the differentials summarized in Fig 4e. This larval filter therefore does not merely depress performance; it redefines larval success under OA as rapid passage through a calcification bottleneck.

**Fig 3:**
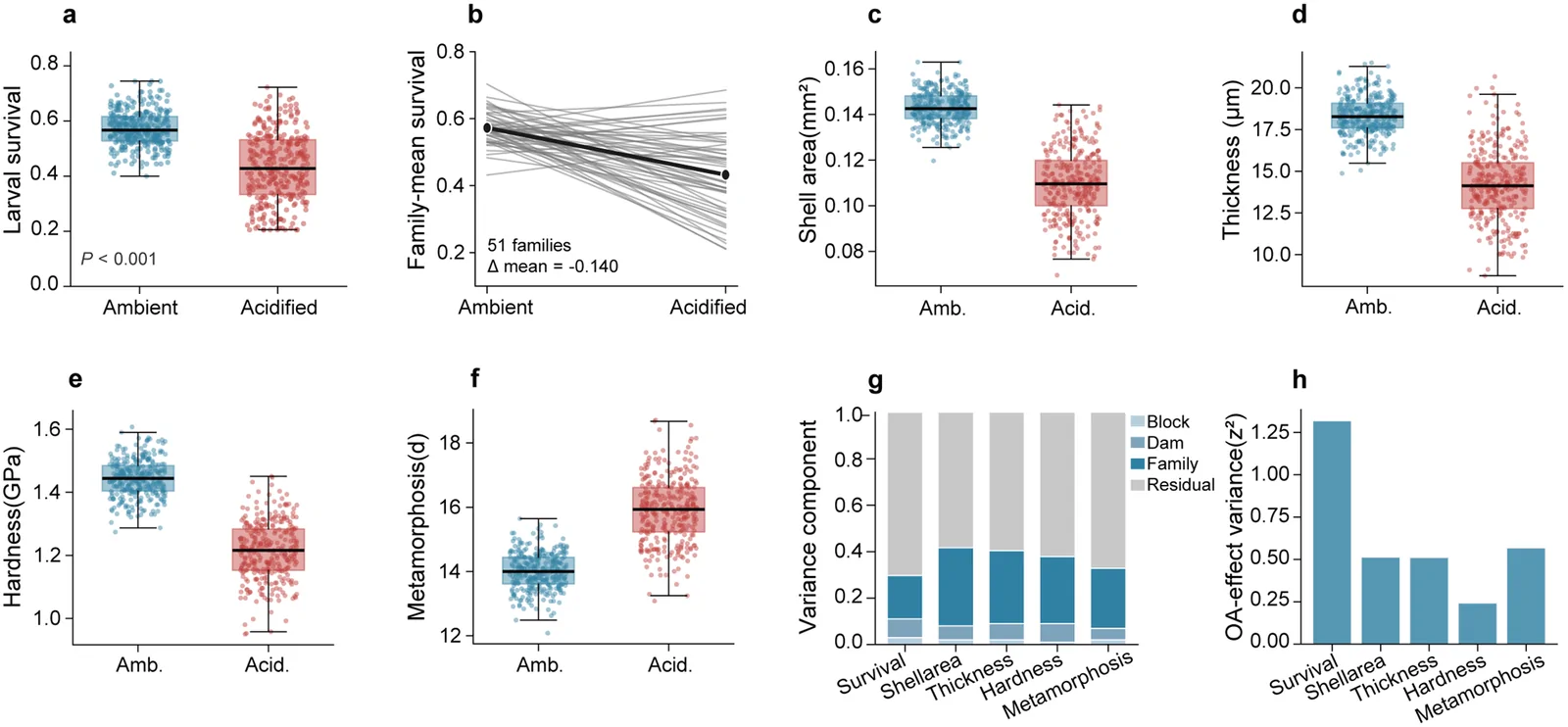
Acidification preserves family variation while narrowing larval performance. **a,** Vial-level larval survival under ambient and acidified seawater. **b,** Family reaction norms showing the mean survival decline under OA and the spread of family-specific responses. **c–f,** OA reduces shell area, shell thickness and shell hardness, and delays metamorphosis. **g,** Pedigree-aware variance partitioning across larval traits. **h,** Among-family variance in standardized OA effect size, showing that acidification retains selectable family differences even while depressing mean larval performance. The numerical data underlying this figure are provided in https://doi.org/10.6084/m9.figshare.34030317.

**Fig 4:**
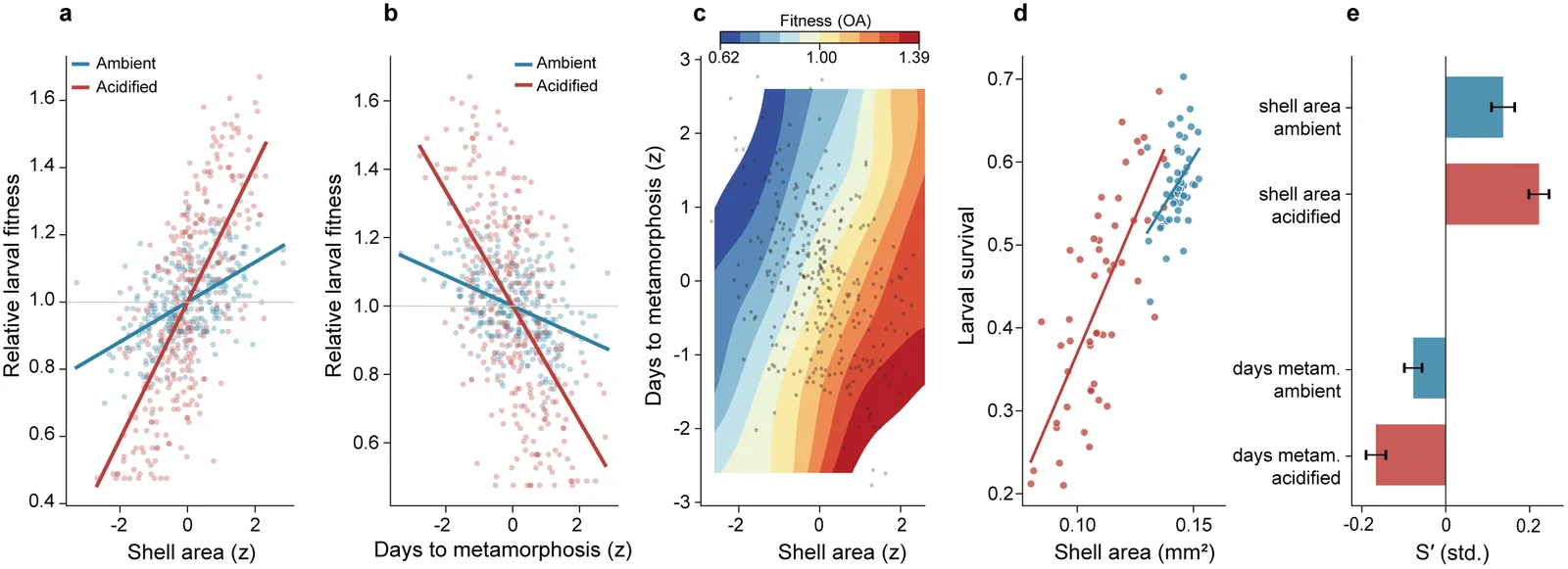
Aidification selects for large first shells and rapid metamorphosis. **a, b,** Relative larval fitness as a function of standardized shell area and days to metamorphosis under ambient and acidified conditions; slopes show stronger OA selection for larger shell area and faster metamorphosis. **c,** Kernel-smoothed two-dimensional OA fitness surface, highlighting the restricted high-fitness region. **d,** Family-level shell area vs. survival by treatment, linking the surface to the raw family phenotype. **e,** Standardized selection differentials (*S*′) with 95% confidence intervals, summarizing how OA steepens the larval selection surface. Fitness in *a–c* is vial-level survival relative to the treatment mean (Materials and methods §6.4). The differentials in *e* are standardized on the pooled data. The numerical data underlying this figure are provided in https://doi.org/10.6084/m9.figshare.34030317.

After metamorphosis, acidification acted on a different physiological accounting system. The juveniles posted a near-unity survival rate over the 90-day nursery, so at this stage acidification is expressed not as mortality but as a maintenance and reproduction cost, and gonadal index measured after two years of common-garden grow-out is the closest available proxy for post-metamorphic fitness. Shell growth declined from 1.395 ± 0.013 to 0.937 ± 0.023 mm month^−1^, dissolution scores rose from 1.07 ± 0.05 to 2.01 ± 0.09, MO_2_ increased by 45%, and gonadal index was still depressed by 26% 2 years later (S13–S16 Tables; Fig 5a–5d). Family-level variance again remained repeatable (ICC_family_ = 0.39 for growth, 0.30 for dissolution, 0.35 for MO_2_ and 0.34 for gonadal index; Fig 5e, 5f), but the axis was no longer the larval one. Within the post-metamorphic stage, the two fitness-proximate traits moved together rather than in opposition: families that retained shell growth under acidification also retained gonadal output (*r* = +0.75, *P* = 2 × 10^−10^; Fig 5g). Growth nevertheless leaves 44% of the variance in reproductive retention unexplained, so it is an incomplete stand-in for post-metamorphic tolerance, and families with the highest metabolic cost under acidification tended to have the lowest delayed gonadal index, although this trend was not statistically significant (*r* = −0.27, *P* = 0.054; Fig 5h). The important contrast is therefore not within the post-metamorphic stage but between stages: family reaction norms make the switch visible, because the families that stand out as larval winners under acidification are not the families that stand out as post-metamorphic winners (S5 Fig). Metamorphosis reweights performance from rapid first-shell construction and developmental tempo to chronic maintenance, dissolution resistance, metabolism, and reproductive allocation.

**Fig 5:**
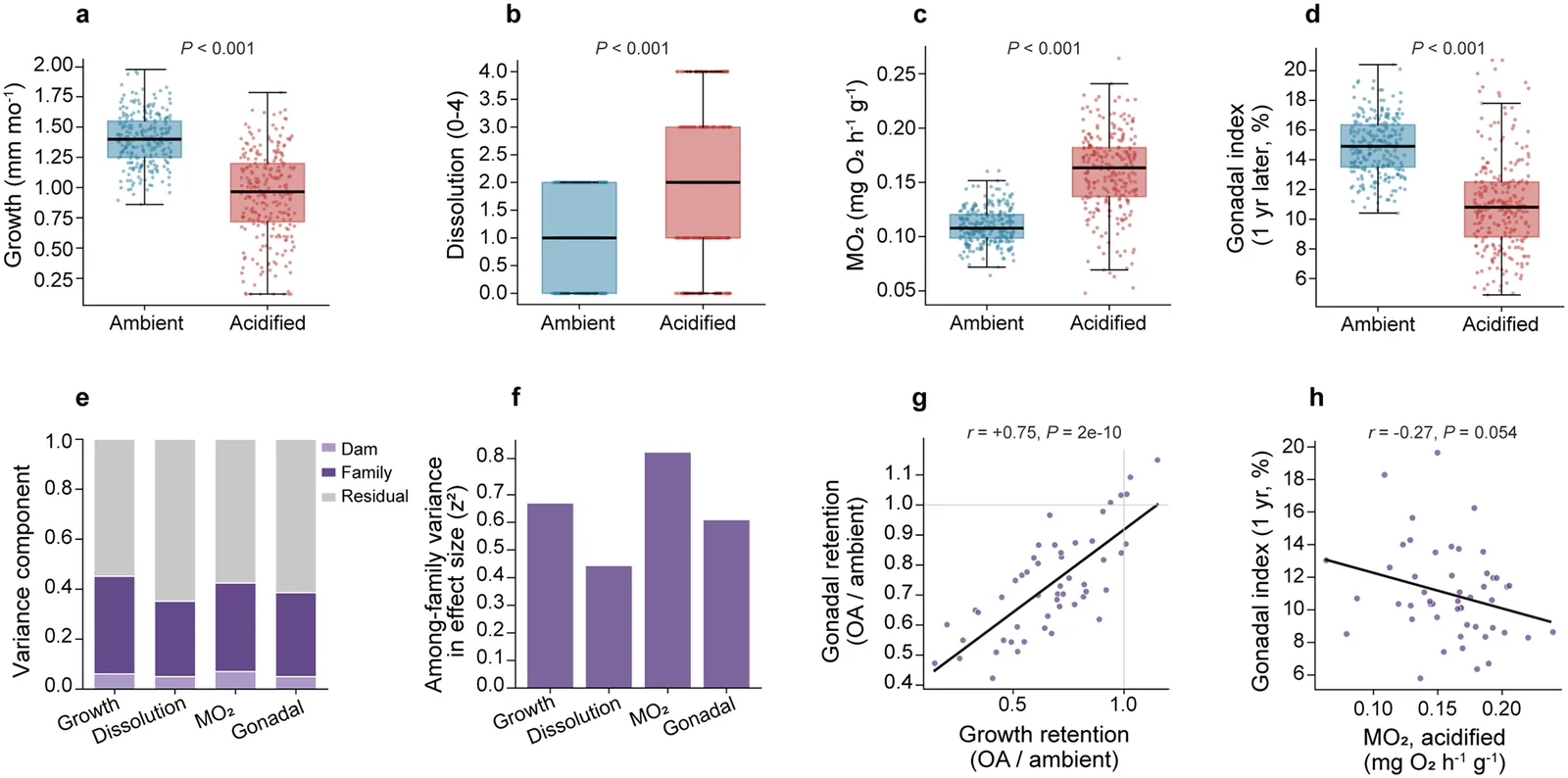
Post-metamorphic acidification exposes a maintenance and reproduction syndrome. **a–d,** Post-metamorphic OA reduces shell growth, increases shell dissolution and MO_2_, and depresses gonadal index measured after 2 years of ambient common-garden grow-out. **e,** Pedigree-aware variance partitioning across adult-stage traits. **f,** Among-family variance in standardized OA effect size, showing that adult-stage tolerance remains repeatable across families. **g,** Family-level growth retention vs. gonadal retention. The two are strongly positively correlated (*r* = +0.75, *P* = 2 × 10^−10^). ***h,*** Family-level MO_2_ under acidification vs. delayed gonadal index; the negative trend is not significant (*r* = −0.27, *P* = 0.054). MO_2_ is the mass-specific rate of oxygen consumption, used here as an index of routine maintenance metabolism. The numerical data underlying this figure are provided in https://doi.org/10.6084/m9.figshare.34030317.

### Acidification reorganizes the multivariate architecture into a cross-stage conflict

Composite scores show that family rankings invert, but not whether the covariance structure itself is responsible, so we estimated the among-family covariance matrix G for all nine traits at once, separately within each environment (Materials and methods, §7.4; S17 and S18 Tables). Two quantities summarize such a matrix. Its leading eigenvector, *g*_max_, is the single weighted combination of the nine traits along which families differ most; the effective number of dimensions, *n*_D_, is the sum of the eigenvalues divided by the largest, and is 1 when all among-family variance lies on *g*_max_ alone and 9 when it is spread evenly across the traits. Under acidification, the architecture is close to one-dimensional: $g_{\text{max}}$ accounts for 57% of the among-family variance (95% CI 49%–66%; $n_{\text{D}}$ = 1.77) and exceeds its design-based null (*P* = 5 × 10^−4^; Fig 2f). Nineteen of the 20 cross-stage correlations are negative, with a mean of −0.375 (95% CI −0.50 to −0.20; *P* = 5 × 10^−4^; Fig 2g). Under ambient conditions, the same 51 families show no such structure: the mean cross-stage correlation is −0.001 (*P* = 0.49; Fig 2h), $g_{\text{max}}$ accounts for only 37% against a null expectation of 35%, and variance is spread over more dimensions ($n_{\text{D}}$ = 2.70). Under acidification $g_{\text{max}}$ loads positively on all five larval traits and negatively on all four post-metamorphic traits, so the dominant axis of family variation is the cross-stage conflict itself, whereas the ambient $g_{\text{max}}$ loads on the post-metamorphic traits without a consistent sign (−0.22 for metabolic efficiency, +0.29 for gonadal index; Fig 2i), so it describes no cross-stage conflict. The contrast is well supported (Δr = −0.374, 95% CI −0.56 to −0.16), and the two matrices differ in orientation as well as dimensionality ($g_{\text{max}}$ 42.5° apart; Krzanowski’s common-subspace statistic 2.28 of a possible 4; mean random-skewers vector correlation 0.66). Acidification does not simply depress performance at both stages—it converts an architecture in which the two stages vary independently into one in which they oppose each other.

The consequence for selection follows directly. Under acidification, the predicted multivariate response to selection on larval fitness, $R\;=\;G\beta_{\text{larval}}$, is positive across the larval traits but negative across the post-metamorphic traits (summed post-metamorphic response −1.91, 95% CI −2.57 to −1.20; S6a Fig; S19 Table), and it points 100° away from the direction that post-metamorphic selection favors—more than a right angle, so the correlated response actively opposes adult-stage selection rather than merely failing to help it. The reciprocal holds (111°), whereas under ambient conditions the same calculation yields no cross-stage cost at all (+0.09; 88°). The conflict axis is not an artifact of nonadditive or maternal effects: among-sire covariances, which isolate the additive component, give a $g_{\text{max}}$ 16.6° from the family-level one (S6b Fig), explaining a similar share of variance (58%) and carrying the same opposing loadings (mean cross-stage *r* = −0.46). An unweighted principal-component decomposition of the nine tolerance ratios recovers the same axis as the a priori weighted composites (*r* = +0.95 and −0.70; S6c Fig), confirming that the weighting in Eqs. 2, 3 is not the source of the result.

### Single-stage selection enriches hidden liabilities across the life cycle

Finally, we asked whether the observed sign of cross-stage covariance is consequential for adaptation, not merely descriptive of present-day family variance. We projected family frequencies for 20 generations using a relative-fitness family-frequency model with Wright–Fisher mean-fitness normalization (Materials and methods, Eqs. 5–8; S20 Table). Because these projections address evolutionary rather than purely plastic responses in a changing ocean [37,38], the model is not an abundance forecast; it isolates how the sign of cross-stage covariance changes evolutionary direction under the same family pool and the same forcing schedule. Under positive and near-zero scenario surfaces, larval-dominant, balanced, and adult-dominant selection all moved trajectories in broadly adaptive directions, differing mainly in which stage improved faster (Fig 6a, 6b, 6d, 6e, 6g, and 6h). Under the empirical conflict surface, however, stage weighting changed direction: larval-dominant selection improved larval tolerance while eroding adult tolerance, adult-dominant selection produced the inverse, and balanced weighting was the only regime that kept both axes net-positive while retaining the most effective family diversity (Fig 6c,6f,6i). Thus, the same family pool carries adaptive information in opposite directions depending on which stage is screened.

**Fig 6:**
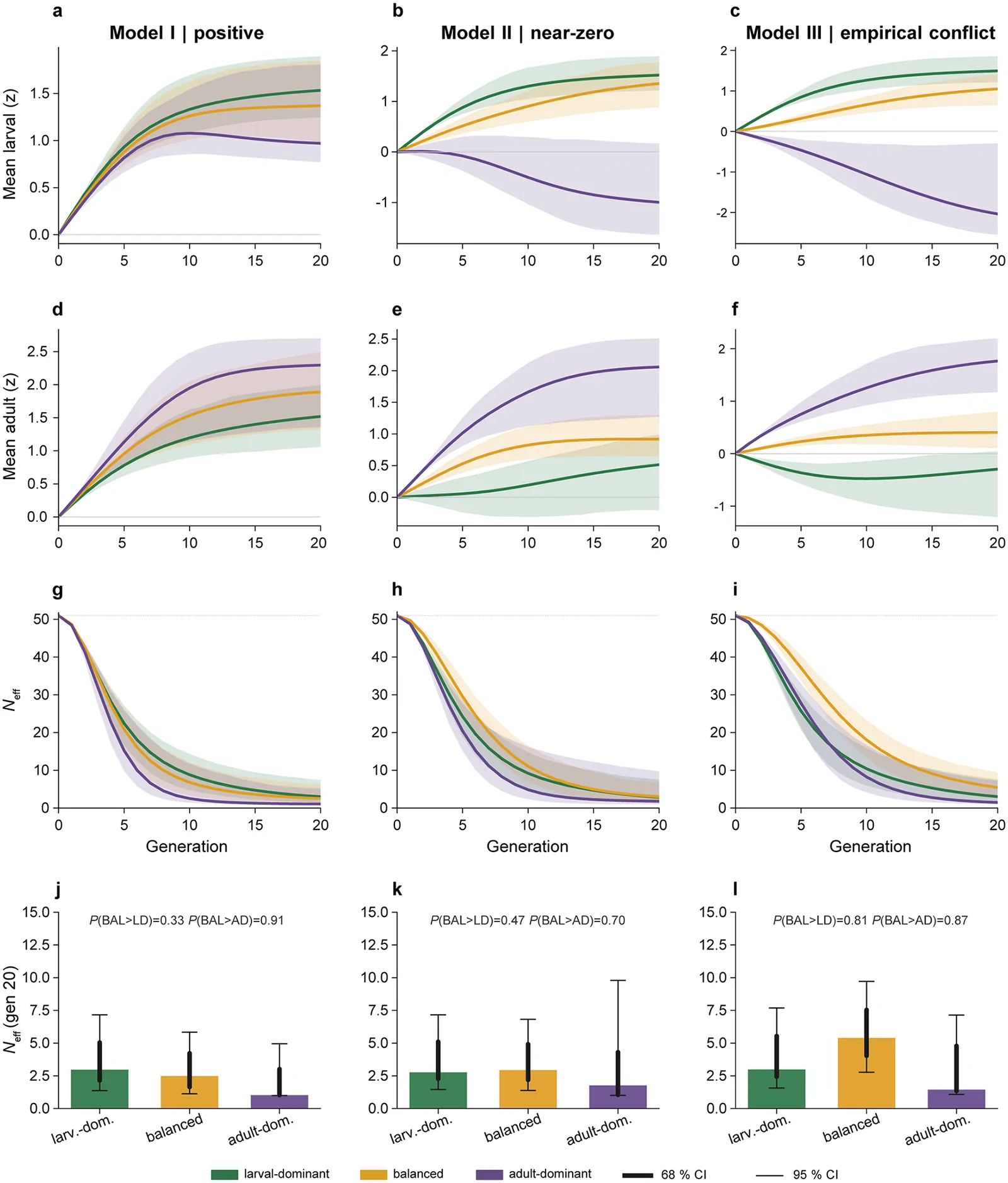
Balanced selection preserves life-cycle resilience when metamorphosis creates genetic conflict. **a–i,** Mean larval tolerance, mean post-metamorphic tolerance and effective family diversity (*N*_eff_) across 20 generations. The y axes of *a–f* are the frequency-weighted means ${\overset{―}{z}}_{\text{L}}(g)$ and ${\overset{―}{z}}_{\text{A}}(g)$ of the standardized family composite scores defined in Eqs. 2, 3 and 8, so 0 is the starting population mean and one unit is one among-family standard deviation. Trajectories are shown under positive, near-zero, and empirical conflict surfaces crossed with larval-dominant, balanced and adult-dominant selection. Under the empirical conflict surface, single-stage selection improves the screened stage while eroding the other. **j–l,** Generation-20 endpoints with 68% and 95% paired family-cluster bootstrap intervals. Balanced weighting is favored only when the empirical cross-stage conflict is present, because it preserves both stage performance and family diversity. Absolute $N_{\text{eff}}$ values fall steeply in every regime because the model contains selection only, with no mutation, recombination, or migration (Materials and methods, §8); the comparison between regimes, not the absolute endpoint, is the quantity of interest. The numerical data underlying this figure are provided in https://doi.org/10.6084/m9.figshare.34030317.

At generation 20, paired family-cluster bootstraps showed that balanced weighting exceeded larval-dominant selection with probability 0.81 and adult-dominant selection with probability 0.87 under the empirical conflict surface (Fig 6j–6l; Eq. 9). These are posterior-style probabilities from paired resamples, not significance tests: 0.81 means that in 81% of bootstrap resamples of the family pool balanced weighting retained more diversity than larval-dominant weighting, which is suggestive rather than decisive, whereas the corresponding probabilities under the positive and near-zero surfaces are 0.33 and 0.47—indistinguishable from a coin toss. The informative comparison is therefore between surfaces, not the absolute value of any one probability. In absolute terms, every regime loses most of the initial diversity by generation 20, because the model deliberately contains only selection: it has no mutation, no recombination among families and no immigration, and families are treated as indivisible units. It is a comparator of evolutionary direction under a common forcing schedule, not a forecast of standing variation, and the differences between regimes should be read as relative rather than absolute—balanced weighting retained ~80% more effective family diversity than larval-dominant weighting and about 3.7-fold more than adult-dominant weighting. Because recombination and mutation would replenish variation in all three regimes, incorporating them would be expected to raise every trajectory without reversing their order, but this remains to be tested. Continuous stage-weight scans placed the diversity maximum near balanced selection ($w_{\text{L}}$ ≈ 0.45; Materials and methods, §8.4), and balanced weighting retained the most diversity in all 36 combinations of forcing intensity, ramp and horizon (S7 and S8 Figs; S21 and S22 Tables). These projections turn the empirical ranking reversal into an operational warning: single-stage selection does not merely omit information from the other stage; when metamorphosis reverses family rankings, it actively enriches hidden liabilities elsewhere in the life cycle.

## Discussion

OA tolerance in *M. mercenaria* is partitioned across life stages rather than integrated across the organism. Family-level larval and adult composites covary negatively; the sign survives every alternative specification, and rank reversals are pervasive. Because the sign of cross-stage covariance dictates the direction of projected evolution, single-stage weighting yields gains in one stage at the cost of the other, and only balanced weighting keeps both trajectories net-positive while preserving family diversity. The multivariate analysis shows that this is a property of the architecture rather than of a scoring rule: under acidification, a single axis carries most of the among-family variance and sets larval against post-metamorphic performance, whereas under ambient conditions that axis does not exist. The covariance geometry is the constraint, not statistical decoration. This sharpens evolutionary-rescue theory [39–42]. Evolutionary-rescue models calibrate rescue against the distance between mean performance and a moving optimum; in a complex life cycle, the fitness axis itself bends between stages. A population rescued on the larval axis can simultaneously lose ground on the adult axis under identical selection—a developmentally structured evolutionary trap that single-stage assays cannot resolve. The most sensitive stage is therefore not the best window onto adaptive capacity: its survivors often carry the largest hidden cost downstream, and single-stage rescue metrics can converge on adaptive futures that are demographically unviable.

Two nonexclusive mechanisms fit the data. First, an energetic trade-off [10,13,21,22]: families that rapidly clear the larval carbonate bottleneck may pay through elevated maintenance metabolism, reduced dissolution resistance and diminished reproductive allocation—a thermodynamic, not genetic, conflict. Second, antagonistic pleiotropy or tight cross-stage linkage [26,28,29]: alleles that favor pH homeostasis, first-shell mineralization and developmental tempo at the larval stage may oppose shell maintenance, chronic acid–base regulation and gonadal investment thereafter. Metamorphosis does not erase cross-stage genetic covariance in marine invertebrates: in the ascidian *Ciona intestinalis*, Aguirre and colleagues [29] likewise recovered a single supported eigenvector of the viability G matrix that spanned the metamorphic boundary and set larval viability against viability on either side of it. Our result extends that finding in two ways—the conflict axis here concerns tolerance of an environmental stressor rather than viability under benign conditions, and it is induced by that stressor, being absent from the same families under ambient conditions. A third possibility we cannot exclude is that the ambient architecture is not truly modular but merely has too little among-family variance in the relevant direction to detect. Our design does not localize the underlying loci, but the sign is biologically expected because larval and adult tolerance recruit partly different physiological axes [9,10,12,13,21,34].

The single-stage paradigm has persisted because its blind spot is invisible by construction: when stages are measured on different pedigrees, the cross-stage covariance sign cannot be recovered. The field has thereby converged on a workflow that systematically under-observes a first-order adaptive constraint, and confidence calibration worsens as within-stage sample size grows. The remedy is matched-stage experiments within a shared pedigree [29,30,43], replicated across source populations and forcing intensities, with the covariance sign reported as a primary outcome rather than an afterthought. The phenomenon is unlikely to be confined to bivalves. Many benthic marine invertebrate species—including most commercially and ecologically important calcifiers—share the relevant architecture: a small, carbonate-limited pelagic larva with a narrow developmental window, followed by a benthic adult with different energetic demands and delayed reproductive bookkeeping [5,7,8,44]. Corals, echinoderms and crustaceans show stage-dependent sensitivities consistent with cross-stage conflict but have rarely been tested for covariance sign within a shared pedigree [9,12,34,35,45]. Our result therefore makes a testable prediction: matched-stage assays in these taxa should frequently recover nonpositive, and in some cases negative, cross-stage covariance, and single-stage forecasts should be biased toward whichever stage was screened. The sharper and more easily falsified half of the prediction concerns induction rather than sign: because the conflict axis reported here is absent from the same families under ambient conditions, matched-stage assays run in a benign and a stressful environment should find among-family variance dispersed across several axes in the benign environment and concentrated onto a single cross-stage axis under stress. That comparison is within reach of any system in which the same families can be phenotyped on both sides of a life-history transition, and the design-based null used here (Materials and methods, §7.4) provides a ready test of it. The same logic should extend to warming and hypoxia [20–22,45].

Currently, most OA-adaptation programmes emphasize early larval traits because they scale cheaply and dominate early mortality [9–15]. Uncorrected, such screening will enrich broodstock that traverse the larval filter while exporting costs to adult maintenance and reproduction; the same logic distorts restoration triage of wild cohorts. Selection indices that integrate stage-specific performance with cross-stage covariance—explicit balanced weighting of the kind implemented here—provide a transparent corrective at low operational cost [20,30]. Because the relevant covariance appears only under acidification, such indices have to be estimated under the stressor rather than inferred from performance in benign conditions. The cost of inaction is a generation of selection pressure applied to the wrong axis. The forecasting consequences are larger. When single-stage collinearity fails, the screened stage does not merely under-sample adaptive capacity; it reports adaptive capacity oriented in the wrong direction. Resilience in complex-life-history taxa is not the variance expressed in the most vulnerable stage, nor the magnitude of the strongest immediate response: it is the subset of variance that remains favorable after the life cycle re-weights performance across stages [46,47]. The critique extends beyond OA to warming, hypoxia, and multi-stressor regimes wherever metamorphosis reorganizes exposure and selection. The result sits in a clear conceptual lineage: OA reshapes within-stage fitness landscapes [15], and evolution of one climate-tolerance trait can carry correlated physiological and life-history costs [48]; here, the geometry operates across entire life stages, and under OA it is antagonistic.

Several limitations define the next steps. We examined one source population at one OA intensity; sign and magnitude may vary with genetic background and forcing gradient [37,38,49]. We also did not test parental or transgenerational acclimation. All comparisons should therefore be interpreted as within-generation family-level tolerance and covariance under a common experimental background, rather than as evidence for or against inherited plasticity across generations. The projections treat family composites as indivisible units of selection and do not model recombination, mutation, migration, or density dependence [40,42]; they therefore describe the direction in which selection pushes a fixed family pool, not the amount of genetic variation a real population would retain, and the absolute endpoint diversities they report should not be read as predictions. Adding these processes is a tractable extension. G itself was estimated at the among-family level on a partial pedigree, so it contains nonadditive and maternal as well as additive variance; the close agreement between the family- and sire-level conflict axes indicates that the additive component carries the same sign and orientation, but resolving their relative magnitudes will require a larger and denser breeding design. Studies setting out to emulate this design should increase sire replication in particular. Twelve sires proved sufficient to confirm the sign and orientation of the conflict axis at the additive level, but constraining the additive genetic variance itself needs a larger paternal array, and that is where additional effort would repay first. The pedigree animal model strengthens inference on covariance sign but does not resolve the full additive-genetic architecture [30,43,50]; localizing the loci, and distinguishing antagonistic pleiotropy from linkage, will require denser pedigrees and scaled genotyping. These caveats locate the next experiments rather than weaken the central message. Resilience is not a trait of a single-stage; it is a distributed property of the developmental sequence, and environmental change can reorganize that distribution in ways that invert winners and losers. What must be measured, and preserved in downstream forecasts, is the covariance structure of performance across the life cycle that selection actually sees.

## Materials and methods

The experimental design, measurement timing, seawater chemistry, and pedigree scaffold are summarized in S1 and S2 Figs and S1–S2 Tables.

### 1. Study organism and broodstock conditioning

Adult *Mercenaria mercenaria* (shell length 62–81 mm; *n* = 130) were collected from a single intertidal–subtidal flat in Long Island Sound, Connecticut, USA, in order to minimize confounding by population structure and to retain a common evolutionary background across the pedigree. Animals were transported to the hatchery and acclimated for 21 d in flow-through, UV-treated seawater (20 ± 0.5 °C; 30 ± 0.5 psu; pH_T_ = 8.05 ± 0.02), and fed ad libitum with *Isochrysis galbana* and *Chaetoceros calcitrans* at a 1:1 ratio (~5 × 10^4^ cells mL^−1^). Shell length, total wet mass, and sex were recorded before crossing.

Spawning was induced by thermal stimulation and was carried out within a restricted temporal window to limit uncontrolled spawning-order effects. Broodstock were strip-spawned only when necessary to complete missing crosses. Embryos from each cross were rinsed through 40-µm mesh, checked for fertilization success after first cleavage, and retained only when cleavage exceeded 80%. This procedure generated the operational family units that were carried consistently through larval assays, juvenile assays, source-data worksheets and pedigree-aware models.

### 2. Quantitative-genetic family design

The pedigree was intentionally partial rather than fully factorial [30]. Each of 12 sires was crossed to a subset of the 6 dams, yielding 51 operational families (S1 Fig). This design sacrifices some balance relative to a complete diallel but preserves the paternal half-sib structure needed for quantitative-genetic inference while remaining practical for a nonmodel bivalve spawning programme. Operational families were mapped to an explicit sire–dam table and then carried through every analysis in one of three ways: as family identifiers, as nested pedigree terms within mixed models, and as the numerator-relationship structure used for the supplementary animal model [30,50]. Families sharing a sire are not independent evolutionary units, so retaining the pedigree in all downstream models prevents spurious certainty in family rankings and provides a conservative framework for evaluating cross-stage covariance.

Because the pedigree is partial and the phenotypic inputs used for the bivariate animal model are family-mean composite scores rather than repeated individual records, the quantitative-genetic analysis is interpreted as a formal support for covariance sign and mode rather than as a definitive estimator of all narrow-sense parameters. This boundary is made explicit in the main manuscript.

### 3. Seawater chemistry manipulation and characterization

Ambient and acidified systems were maintained independently and continuously monitored following established ocean acidification best-practice workflows [51]. Ambient seawater targeted present-day local conditions, whereas the acidified treatment targeted end-century carbonate chemistry under a high-emissions trajectory (pH_T_ ≈ 7.70, Ω_arag_ ≈ 1.0). Independent treatment loops were used to avoid pseudo-replication at the chemistry level and to preserve treatment-specific carbonate-system variance for downstream mixed-model inference.

pH was logged continuously with NBS-calibrated electrodes and independently checked by benchtop spectrophotometric measurements. Total alkalinity was measured regularly by open-cell titration, and carbonate-system variables were recalculated from pH and total alkalinity with CO2SYS [52], following best-practice carbonate chemistry reporting [53]. Temperature and salinity were recorded daily. Treatment separation and its stability over the exposure are shown in S2 Fig, and the full carbonate-system record is summarized in S1 Table. Treatment systems were serviced separately to avoid gas-exchange cross-talk, and water-exchange rates were adjusted to keep dissolved oxygen, temperature, and salinity tightly overlapping between treatments.

### 4. Larval experiment: Hierarchical design and phenotyping

The larval assay was designed to separate treatment effects from temporal and vessel effects. Two temporal blocks were staggered by 5 d. Each family was reared in its own independently plumbed 20-L polycarbonate tank within each treatment × block combination (51 families × 2 treatments × 2 blocks = 204 tanks), and each tank contained three 250-mL larval vials. Each vial received exactly *N*_*0*_ = 180 larvae from a single family, counted individually under stereomicroscopy, giving 51 × 2 × 2 × 3 = 612 vials in total (S1 Data). Larval and post-metamorphic exposures were entirely independent of one another: no animal experienced acidification at both stages. Larvae used in the larval assay were terminated at that assay’s endpoint, and the juveniles entering the post-metamorphic experiment were drawn from separate ambient-reared cohorts of the same 51 families and then randomly assigned to the ambient or acidified nursery. Larval and post-metamorphic tolerance are therefore linked only through shared family (and hence pedigree) membership, which is precisely the level at which selection operates and at which cross-stage covariance is identifiable; no result in this paper depends on within-individual carry-over.

Larvae were reared under constant gentle aeration and fed every 48 h with *I. galbana* plus *C. calcitrans* with a 1:1 mixture at 10^4^ cells mL^−1^. Vials were terminated when at least 50% of survivors reached the pediveliger stage, or at day 21 if that threshold had not yet been reached. All surviving larvae were counted, and dead individuals were identified by collapsed or darkened tissue. From each vial, up to 20 survivors were sampled for shell area (calibrated image analysis), shell thickness (cross-sectional SEM), nanoindentation hardness (Hysitron TI950; Berkovich probe; 500 µN peak load; 5-s hold) and days to metamorphosis. Image analysis, indentation, and SEM scoring were performed blind to family and treatment identity.

### 5. Adult experiment: Phenotyping and delayed reproductive signature

The adult phase was designed to capture both acute post-metamorphic maintenance costs and a delayed reproductive signature of earlier OA exposure. Therefore, our designation of “adult” stage includes reproductively active adults as well as post-metamorphic juveniles. Five juveniles per family were assigned to each treatment (*n* = 510 total individuals) and held individually in partitioned chambers nested within flow-through treatment tanks during a 90-d OA nursery phase.

Shell growth rate was calculated from repeated caliper measurements of maximum shell length over the nursery interval and is reported as mm per 30-d period. Shell dissolution and shell growth are not alternatives: in bivalves the periostracum-covered growing margin continues to accrete new carbonate while the older, abraded umbonal and lateral surfaces—where the periostracum is thin or worn—are exposed to undersaturated water and corrode. Net linear growth and localized surface dissolution therefore proceed simultaneously on the same shell, and both were scored. Dissolution was quantified on the external shell surface as a 0–4 ordinal scale (0 = intact periostracum and no visible etching; 1 = localized loss of surface luster; 2 = visible etching or chalky patches confined to the umbo; 3 = etching extending over the lateral surfaces with exposed prismatic layer; and 4 = extensive pitting or perforation of the outer layer) by two trained scorers working independently and blind to family and treatment identity (Cohen’s *κ* = 0.83); disagreements were resolved by consensus. Oxygen consumption (MO_2_)—the mass-specific rate of oxygen uptake, used here as an index of routine maintenance metabolism—was measured by sealed-chamber respirometry using a YSI ProODO optical O_2_ logger during 3-h incubations at days 30, 60, and 90; rates were standardized to dry mass. Nearly all juveniles survived the 90-day nursery (survival rate ≈ 1), so post-metamorphic fitness variation among families is expressed through growth, shell maintenance, metabolic cost and subsequent reproductive output rather than through differential mortality.

After the OA nursery phase, all juveniles were transferred back to ambient pond culture. Gonadal index was then quantified after two years of common grow-out as (gonad wet mass/total soft-tissue wet mass) × 100%, so reproductive status is interpreted as a delayed signature of earlier OA exposure rather than a trait measured concurrently with the 90-d maintenance phenotypes. This timing distinction is preserved throughout the analysis and the discussion.

### 6. Statistical analyses

For each trait, we first screened biologically justified random-effect structures and then estimated treatment effects using the most conservative supported structure. Candidate random terms followed the mating design and the experiment hierarchy: sire, dam, operational family, and block for larval traits; sire, dam, and operational family for adult traits. The objective was not to maximize model complexity but to preserve pedigree and block structure whenever it remained estimable and informative. All analyses were implemented in Python with pandas, numpy, scipy, statsmodels, openpyxl and matplotlib, following mixed-model logic commonly used in lme4 and glmmTMB workflows [54,55].

#### 6.1 Larval survival (pedigree-aware mixed model).

Larval survival was analyzed as a pedigree-aware mixed model on the empirical-logit transform [56] of vial-level survival proportions:


$$\begin{array}{c} {logit}^{*}(p_{i})=log(\frac{p_{i}+\frac{\text{1}}{\text{2}n_{i}}}{\text{1}-p_{i}+\frac{\text{1}}{\text{2}n_{i}}}), \end{array}$$

where $p_{i}$ is the survival proportion and $n_{i}$ the initial vial stocking. The response was modeled as a function of treatment while retaining the supported pedigree and block structure:


$$\begin{array}{c} {logit}^{*}(p_{i})=\mu +\beta \cdot T_{i}+u_{block(i)}+u_{sire(i)}+u_{dam(sire,i)}+u_{family(i)}+\varepsilon_{i}, \end{array}$$

with $T_{i}$ a treatment indicator (0 = ambient, 1 = acidified), β the fixed effect of acidification, each u. a Gaussian random effect and $\varepsilon_{i}$ a Gaussian residual. This transformation stabilizes variance, avoids boundary infinities and yields effect sizes that remain interpretable on the logit scale.

#### 6.2 Continuous larval and adult traits (linear mixed models).

Continuous larval and adult traits were analyzed with pedigree-aware linear mixed models [54]. We did not force a single identical random structure across traits, because doing so would privilege formal symmetry over biological fidelity and numerical stability. Instead, each trait retained the richest nonsingular structure supported by the observed data distribution, as recorded for that trait in the statistical supplement.

#### 6.3 Intraclass correlation.

For each trait y modeled with random sire, dam and family terms nested in a block hierarchy,


$$\begin{array}{c} y=\mu +\tau_{treat}+u_{block}+u_{sire}+u_{dam(sire)}+u_{family}+\varepsilon_{i}, \end{array}$$

the intraclass correlation attributed to family is


$$\begin{array}{c} {ICC}_{family}=\frac{\sigma_{family}^{\text{2}}}{\sigma_{block}^{\text{2}}+\sigma_{sire}^{\text{2}}+\sigma_{dam(sire)}^{\text{2}}+\sigma_{family}^{\text{2}}+\sigma_{\varepsilon }^{\text{2}}}. \end{array}$$

Values are reported with the corresponding larval and post-metamorphic variance-partitioning results in S7–S11 and S13–S16 Tables.

#### 6.4 Selection-surface analysis (Lande–Arnold).

Vial-level relative fitness was defined within each treatment t as


$$\begin{array}{c} w_{i}=\frac{S_{i}}{{\bar{S}}_{t}}\;, \end{array}$$

where *S*_*i*_ is the survival proportion in vial *i* and *S*_*t*_ is the treatment-mean survival, so that vial-level relative fitness is survival expressed relative to the average vial in the same treatment; values *w*_*i*_ > 1 identify vials whose larval survival exceeded the treatment-average expectation. Relative survival defined this way is the fitness measure used in every larval selection analysis reported below. Trait values were standardized to zero mean and unit variance across the pooled data, so that the ambient and acidified estimates are expressed on a common yardstick and are directly comparable, while relative fitness itself is computed within treatment. Eq. 1 gives the full second-order form of the Lande–Arnold model [17,36]. The quantities reported in S12 Table are standardized selection differentials *S*′: trait-by-trait ordinary-least-squares regressions of *w* on each standardized trait, fitted separately within each treatment, together with the retained quadratic term for days to metamorphosis. They measure total selection on each trait; the multivariate selection gradients *β* used for the predicted responses R = Gβ are estimated and reported with the multivariate analysis (Materials and methods §7.4).


$$\begin{array}{c} w_{i}=\alpha_{t}+\beta_{\text{1}}^{\prime }z_{\text{1},i}+\beta_{\text{2}}^{\prime }z_{\text{2},i}+\frac{\text{1}}{\text{2}}\gamma_{\text{1}\text{1}}^{\prime }z_{\text{1},i}^{\text{2}}+\frac{\text{1}}{\text{2}}\gamma_{\text{2}\text{2}}^{\prime }z_{\text{2},i}^{\text{2}}+\gamma_{\text{1}\text{2}}^{\prime }z_{\text{1},i}z_{\text{2},i}+\varepsilon_{i}, \end{array}$$ (1)

Here $z_{\text{1},i}$ denotes standardized shell area, $z_{\text{2},i}$ standardized days to metamorphosis, $\alpha_{t}$ a treatment-specific intercept, $\beta_{\text{1}}^{\prime }$ and $\beta_{\text{2}}^{\prime }$ directional gradients,$\gamma_{\text{1}\text{1}}^{\prime }$,$\gamma_{\text{2}\text{2}}^{\prime }$,$\gamma_{\text{1}\text{2}}^{\prime }$ nonlinear or correlational gradients, and $\varepsilon_{i}$ a Gaussian residual.

### 7. Cross-stage family association and composite tolerance

Cross-stage analyses were based on stage-specific composite retention scores rather than on raw trait deltas, so that resilience is captured as stage-wide performance under stress rather than success in any one endpoint. For each family f, larval composite tolerance$\;C_{f,L}$ and adult composite tolerance $C_{f,A}$ were calculated as weighted geometric means of trait-retention ratios: Family composite scores, rank-reversal diagnostics, and robustness checks are summarized in S3–S5 Tables.


$$\begin{array}{c} C_{f,L}=\prod_{k\in L}{\left(R_{f,L,k}\right)}^{\omega_{L,k}},C_{f,A}=\prod_{k\in A}{\left(R_{f,A,k}\right)}^{\omega_{A,k}}. \end{array}$$ (2)

The multiplicative form preserves multiplicative penalties: a family cannot appear highly tolerant overall if it performs very poorly on any one key component trait. The equivalent log-form used as the regression target is


$$\begin{array}{c} ogC_{f,L}=\sum_{k\in L}\omega_{L,k}log\;R_{f,L,k},log\;C_{f,A}=\sum_{k\in A}\omega_{A,k}log\;R_{f,A,k}. \end{array}$$ (3)

Default stage-specific weights were assigned a priori according to each trait’s expected proximity to fitness. Larval tolerance weighted survival most strongly (0.60), followed by metamorphosis rate (0.20), because these traits define passage through the pelagic demographic bottleneck; shell area, thickness, and hardness received smaller weights (0.08, 0.07, and 0.05) as mechanistic components of early calcification. Post-metamorphic tolerance weighted growth (0.45) and delayed gonadal index (0.35) most strongly as proxies for somatic performance and reproductive allocation, with dissolution resistance and metabolic efficiency retained as supporting maintenance-cost traits (0.10 each). Five alternative weighting schemes produced the same negative cross-stage association, indicating that the result was not driven by the default weighting choice (S5 Table; S3 Fig).

#### 7.1 Effect sizes, confidence intervals and sensitivity.

Robustness of the cross-stage signal was evaluated in four ways: Pearson and Spearman correlation on the observed family composites; pedigree-conditioned residual correlation after removing sire and dam structure by OLS; alternative composite definitions and trait subsets; and threshold sweeps that asked whether the sign changed when only the top-ranked fraction of families was emphasized. These sensitivity analyses are archived in S4–S5 Tables and S3 Fig. Composite-score associations were accompanied by family-cluster bootstrap confidence intervals [57].

#### 7.2 Bivariate pedigree animal model.

As a formal quantitative-genetic sensitivity analysis, we fitted a bivariate pedigree animal model to larval and adult composite tolerance. Standardized family composite scores were stacked into a two-trait response vector, fixed effects were limited to an intercept for each trait, and additive covariance was induced through the numerator-relationship matrix A implied by the partial pedigree, following standard animal model logic [43,50]:


$$\begin{array}{c} y=X\beta +Za+e,a\sim N(\text{0},A⊗G),e\sim N(\text{0},I⊗R). \end{array}$$ (4)

Here y is the stacked two-trait response vector, X and Z are the fixed- and random-effect design matrices, β the vector of fixed effects, a the additive-genetic effect, e the residual term, G the 2 × 2 additive-genetic variance–covariance matrix, R the 2 × 2 residual variance–covariance matrix, and the additive cross-stage genetic correlation is


$$\begin{array}{c} r_{G}=\frac{G_{LA}}{\sqrt{G_{LL}G_{AA}}}. \end{array}$$

The model was fitted by restricted maximum likelihood (REML; [58]), and the profile-supported region of *r*_*G*_ was characterized by the likelihood-ratio threshold 2Δ_REML_ = 3.84 (S6 Table; S4 Fig). Because the fitted phenotypes are family means on a partial pedigree, the model is interpreted as quantitative-genetic support for the direction of cross-stage covariance rather than as a complete resolution of the pleiotropic architecture.

#### 7.3 Sire- and dam-level quantitative-genetic summaries.

To complement the animal model, family composite scores were collapsed separately to shared sires and shared dams. For each sire or dam, larval and adult composite tolerance were averaged across associated operational families and the cross-stage association was quantified with Pearson correlation and Fisher-z confidence limits (S6 Table; S4 Fig). The sire-level summary is especially informative because sires define the shared paternal half-sib structure retained in the pedigree [30].

#### 7.4 Multivariate among-family covariance matrix (G).

The bivariate animal model of Eq. 4 answers the primary, directional question most powerfully, but a full nine-trait additive G is not identifiable on this pedigree (45 covariance parameters from 51 family means). That limit applies to the additive matrix, not to the among-family matrix, which the balanced within-stage replication does support. We therefore also estimated the among-family covariance matrix G for nine traits—five larval (survival on the empirical-logit scale, shell area, thickness, hardness and developmental rate, the reciprocal of days to metamorphosis) and four post-metamorphic (shell growth, dissolution resistance, metabolic efficiency, and gonadal index)—separately within each environment, with every trait oriented so that larger values are better and standardized to unit variance. Because the design is balanced, G follows in closed form from a nested analysis of covariance: the within-stage blocks are $\frac{\left({\text{MS}}_{\text{among}}\;-\;{\text{MS}}_{\text{within}}\right)}{k}$, and the cross-stage block is the covariance of the larval and post-metamorphic family means, which is unbiased because the two stages were measured on different animals. MS denotes a mean square—the among-family and within-family mean cross-product matrices—and *k* is the number of replicates per family per treatment: six vials for the larval traits and five juveniles for the post-metamorphic ones. Matrices were bent to the nearest positive-definite matrix before eigen-decomposition.

Uncertainty came from family-cluster bootstrapping (B=3,000). The bootstrap resamples the 51 families with replacement, each draw taking a family’s whole set of vials and juveniles together so that the resampling unit is the unit of inference; G is re-estimated from scratch within every draw and every derived statistic recomputed from it, and the intervals reported are the 2.5th and 97.5th percentiles of the resulting distributions. Support was assessed against a design-based null—2,000 simulations with the cross-stage covariance set to zero and every other feature of the design preserved—which propagates the sampling error of the estimator itself [59]; effective dimensionality is reported as *n*_D_, the sum of the eigenvalues divided by the largest [60], so that *n*_D_ = 1 would mean all among-family variance lies on a single axis. Each eigenvector of G is a weighted combination of the nine traits and is read as a pattern rather than as a set of separate effects: traits loading with the same sign vary together among families, traits loading with opposite signs vary in opposition, and the size of a loading is that trait’s contribution to the axis, while the associated eigenvalue is the amount of among-family variance lying along it. It is the sign structure of *g*_max_, not its magnitude, that makes the leadin*g* axis a cross-stage conflict rather than a general axis of vigour. The two environments were compared in three ways. The angle between their leading eigenvectors measures how far the dominant axis of family variation has rotated. Krzanowski’s common-subspace statistic [61] asks how much of the space spanned by the four leading eigenvectors of one matrix is also spanned by the four leading eigenvectors of the other, on a scale running from 0 for two entirely distinct subspaces to 4 for two identical ones. Random skewers draws 5,000 random unit selection vectors, projects each through both matrices and records the correlation between the two predicted responses, so that a mean well below 1 means the same selection pressure would be redirected differently by the two matrices. To express the consequence for selection, we estimated stage-specific gradients under acidification—$\beta_{\text{larval}}$ from vial-level relative survival and $\beta_{\text{adult}}$ from family-level relative gonadal index, the appropriate fitness proxy at a stage where survival was near-unity—and evaluated the predicted response R = Gβ. We report its direction rather than an absolute rate, because family means carry nonadditive and maternal as well as additive variance; the decomposition was therefore repeated on among-sire covariances. Because a change in G can arise either from a change in the correlations among traits or from a change in how much among-family variance there is to distribute, both are reported separately. G is deposited in its correlation form in S17 Table, the among-family variance of each trait in each environment in S23 Table, and the eigen-analysis is repeated on the correlation scale so that the two can be told apart. Retaining the family-level is deliberate: nonadditive variance contributes materially to adaptive potential under environmental change in marine invertebrates [62], and the family is the unit that hatchery and restoration programmes actually select.

### 8. Family-frequency evolutionary projection

To project evolutionary trajectories under end-century OA, we implemented a relative-fitness family-frequency model [63] in which each of the *N* = 51 families occupies a point in larval–adult tolerance space and frequencies are updated by Wright–Fisher mean-fitness normalization [63]. Projection scenarios differed in two respects: the geometry of the cross-stage landscape (Model I, positive association; Model II, near-zero association; Model III, empirical conflict) and the relative weighting assigned to larval versus adult performance. Projection outputs are tabulated in S20–S22 Tables.

Initial frequencies are uniform, $p_{f}(\text{0})=\text{1}/N$. The forcing ramp is


$$\begin{array}{c} \alpha (g)=\alpha_{\text{0}}+\beta g, \end{array}$$ (5)

with $\alpha_{\text{0}}$ = 0.22 and β = 0.02 per generation as defaults. Under stage weights ($w_{L}$, $w_{A}$) constrained to $w_{L}+w_{A}\;=\text{1}$, the family-specific selection coefficient is


$$\begin{array}{c} s_{f}(g)=\alpha (g)[w_{L}z_{L,f}+w_{A}z_{A,f}]. \end{array}$$ (6)

Here $z_{L,f}$ and $z_{\text{A},\text{f}}$ are family f’s larval and post-metamorphic composite tolerance scores from Eqs. 2, 3, each standardized across the 51 families to zero mean and unit variance: a value of zero denotes a family of average tolerance for its stage, positive values a family more tolerant than average, and one unit is one among-family standard deviation. The stage weights $w_{\text{L}}$ and $w_{\text{A}}$ set how much each stage contributes to selection and are constrained to sum to one.

Relative fitness is $W_{f}(g)=exp(s_{f}(g)),$ and the family-frequency update follows Wright–Fisher mean-fitness normalization:


$$\begin{array}{c} p_{f}(g)=\frac{p_{f}(g-\text{1})W_{f}(g)}{\sum_{k}p_{k}(g-\text{1})W_{k}(g)}, \end{array}$$ (7)

Population-level outcomes reported at each generation are the frequency-weighted means of the stage-specific composites, ${\overset{―}{z}}_{\text{L}}(g)\;=\;\sum_{\text{f}}p_{\text{f}}(g)\;z_{\text{L},\text{f}}$ and ${\overset{―}{z}}_{\text{A}}(g)\;=\;\sum_{\text{f}}p_{\text{f}}(g)\;z_{\text{A},\text{f}}$—the quantities reported for each generation in the Results, so that 0 is the starting population mean and one unit is one among-family standard deviation—together with the effective family diversity $N_{\text{eff}}$:


$$\begin{array}{c} {\bar{z}}_{L}(g)=\sum_{f}p_{f}(g)z_{L,f},\;{\bar{z}}_{A}(g)=\sum_{f}p_{f}(g)z_{A,f},\;N_{eff}(g)={\left(\sum_{f}p_{f}(g{)}^{\text{2}}\right)}^{-\text{1}}. \end{array}$$ (8)

#### 8.1 Scenario construction.

All three scenarios share the observed larval composite vector $z_{L}$. The adult composite vector is either the empirical observation (Model III) or a realization that shares the marginal variance of $z_{A}$ with an imposed cross-stage correlation ρ=+0.55(Model I) or ρ=0(Model II):


$$\begin{array}{c} z_{A}^{scen}=z(\rho z_{L}+\sqrt{\text{1}-\rho^{\text{2}}}\varepsilon ),\varepsilon \sim N(\text{0},I), \end{array}$$

where (·) denotes the re-standardization operator. Re-standardization after the linear combination ensures comparability across scenarios.

#### 8.2 Forcing-parameter calibration.

The base intensity *α* = 0.22 and ramp *β* = 0.02 were fixed such that, under the empirical surface and balanced weighting, generation-20 $N_{\text{eff}}$ under the empirical surface is ≈ 5.4. Sensitivity across *α* ∈ {0.16, 0.22, 0.28}, *β* ∈ {0.01, 0.02, 0.03} and horizons of 15, 20, 25, and 30 generations is summarized in S22 Table. The qualitative contrast between balanced weighting and one-stage optimization is preserved across the entire grid.

#### 8.3 Paired family-cluster bootstrap uncertainty.

Endpoint uncertainty was quantified by family-cluster bootstrapping with B = 3,000 replicates. The 51 families are resampled with replacement, *z*-scores are recomputed within each resample, and the deterministic projection is rerun under every scenario × weighting combination. Marginal 95% and 68% CIs are read off percentile ranges of the resulting $N_{\text{eff}}$ distribution. Because the same family resamples is applied across weighting regimes, paired-difference probabilities are directly interpretable:


P^(NeffBAL>NeffLD)=1B∑b=1B1[Neff(b),BAL(G)>Neff(b),LD(G)], (9)

We prefer paired bootstrap intervals to per-generation significance tests because adjacent generations in the deterministic projections are not statistically independent.

#### 8.4 Continuous stage-weight scan.

The three anchor regimes (larval-dominant $w_{\text{L}}$ = 0.9, balanced $w_{\text{L}}$ = 0.5, adult-dominant $w_{\text{L}}$ = 0.1) are convenient reference points, not special values. They stand for three selection policies a hatchery or restoration programme could actually adopt: screen almost entirely on larval performance, weight the two stages equally, or screen almost entirely on post-metamorphic performance. In Fig 6, each regime keeps one color throughout; the three columns of panels a–i are the three cross-stage landscape geometries (positive, near-zero and empirical conflict) and the three rows are the three outcomes (mean larval tolerance, mean post-metamorphic tolerance and effective family diversity), so a column is read as one landscape and a row as one outcome, with panels j–l giving the generation-20 endpoints. To check that nothing depends on that choice we re-ran the entire projection across the full larval-weight gradient, from $w_{\text{L}}$ = 0 to $w_{\text{L}}$ = 1 in steps of 0.05, holding $w_{\text{A}}$ = 1 − $w_{\text{L}}$, and recorded the generation-20 outcome at each of the 21 points. The value quoted in the Results as $w_{\text{L}}$ ≈ 0.45 is simply the point on that curve at which generation-20 effective family diversity is maximized under the empirical conflict surface; it is read off the scan, not estimated from a model, and the curve is flat enough near its maximum that any weighting between roughly $w_{\text{L}}$ = 0.40 and $w_{\text{L}}$ = 0.50 gives a closely similar outcome (S21 Table).

Raw vial-level larval data and individual-level post-metamorphic records underlying these analyses are provided in S1 and S2 Data.

## Supporting information

S1 Fig Experimental design and measurement timing. *a*, Life cycle of the hard clam, separating the planktonic larval stage from the benthic post-metamorphic stage. *b*, Schematic of broodstock sourcing, the partial half-sib pedigree, the larval vial-level assay, the post-metamorphic ocean acidification (OA) nursery, and the subsequent two-year common-garden ambient pond grow-out. (TIF)

S2 Fig Seawater chemistry fidelity and treatment stability. ***a,*** Time series of pH_T_. ***b,*** Aragonite saturation state Ω_arag_. ***c,*** Temperature. ***d,*** Salinity. Dotted horizontal lines in a and b indicate treatment targets. Treatment separation was stable throughout the exposure, drift was minimal, and the OA manipulation was imposed under tightly controlled physicochemical conditions. The numerical data underlying this figure are provided at https://doi.org/10.6084/m9.figshare.34030317.
(TIF)

S3 Fig The negative cross-stage association is robust to tolerance definitions and pedigree groups. ***a,*** Pearson *r* and Spearman *ρ* across the baseline and five alternative composite-scoring schemes; the negative sign is invariant to reasonable redefinitions. ***b,*** Equal-weight log-composite scatter. ***c,*** Threshold-quantile sweep showing the fraction of top-quantile families in each stage that fall below the opposite-stage median across quantile cut-offs. ***d,*** Pearson *r* after leave-one-out removal of each sire and each dam, relative to the full-data estimate (dashed vertical line). The numerical data underlying this figure are provided in https://doi.org/10.6084/m9.figshare.34030317.
(TIF)

S4 Fig Quantitative-genetic summaries support a negative cross-stage covariance. ***a,*** Sire-level mean larval versus adult composite tolerance for the 12 shared sires (Pearson *r* = −0.66, 95% CI [−0.90, −0.14], *P* = 0.019). ***b,*** Summary of covariance-sign diagnostics across family-level, pedigree-conditioned, sire- and dam-level, and animal model estimates; every summary retains a negative sign. ***c,*** Restricted-likelihood profile across candidate *rG* values; the dashed horizontal line marks the 2Δ_REML_ = 3.84 threshold. The numerical data underlying this figure are provided in https://doi.org/10.6084/m9.figshare.34030317.
(TIF)

S5 Fig Family reaction norms reveal the stage-specific redistribution of OA performance. **a–d,** Ambient-to-acidified reaction norms for 51 families across representative larval traits (a, survival; *b,* shell area) and adult traits (c, growth; *d,* delayed gonadal index). Families highlighted in red in a and b are the 10 top-quartile larval families that drop below the post-metamorphic median; families highlighted in red in c and d are the 11 top-quartile post-metamorphic families that drop below the larval median. These are the two sets ringed in red in Fig 2b, so the highlighted families differ between the left-hand (larval) and right-hand (post-metamorphic) panels. These reaction-norm panels show the stage-specific redistribution of performance that underlies the cross-stage ranking reversal. The numerical data underlying this figure are provided at https://doi.org/10.6084/m9.figshare.34030317.
(TIF)

S6 FigSupporting detail for the cross-stage covariance analysis.***a,*** Predicted multivariate response to selection on larval fitness, $R\;=\;G\beta_{\text{larval}}$, summed within each stage, under the acidified and the ambient matrix; vertical bars are 95% family-cluster bootstrap intervals (B= 3,000 resamples of the 51 families), and *P*(<0) is the bootstrap probability that the post-metamorphic sum is negative. ***b,*** Trait loadings on the leading eigenvector of the among-sire (paternal half-sib) covariance matrix against the family-level loadings; the two axes are 16.6° apart, so the conflict axis is present in the additive component. ***c,*** Family scores on an unweighted principal component of the nine trait-specific tolerance ratios against the a priori weighted composites, with least-squares fits (*r* = +0.95 and −0.70), showing that the weighting scheme of Eqs. 2 and 3 is not the source of the result. The numerical data underlying this figure are provided at https://doi.org/10.6084/m9.figshare.34030317.(TIF)

S7 FigProjected outcomes are sensitive to continuous stage weighting.***a,*** Generation-20 mean larval tolerance across the full larval-weight gradient. ***b,*** Generation-20 mean adult tolerance. ***c,*** Effective family diversity. Under the empirical conflict surface, the diversity maximum is located near balanced weighting ($w_{\text{L}}$ ≈ 0.45) and drops off on both sides, confirming that the superiority of balanced weighting does not depend on the three anchor scenarios displayed in Fig 6. The numerical data underlying this figure are provided in https://doi.org/10.6084/m9.figshare.34030317.(TIF)

S8 Fig Projection outcomes are robust to forcing design. ***a–c,*** Generation-20 effective family diversity against generation horizon under a base forcing intensity of *α*_0_ = 0.16 (*a*), 0.22 (*b*) and 0.28 (*c*), for each of the three stage weightings. Line color encodes the weighting; line style encodes the per-generation ramp *β*. Absolute values vary with forcing design, but balanced weighting retains the most family diversity in all 36 combinations of *α*_0_, *β,* and horizon (S22 Table). The numerical data underlying this figure are provided in https://doi.org/10.6084/m9.figshare.34030317.
(TIF)

S1 Table Seawater chemistry records and treatment fidelity. Chemistry record, sampled every third day across the 91-day exposure (31 sampling days per treatment), underlying the treatment descriptions in the manuscript.
(XLSX)

S2 Table Partial half-sib pedigree design. The partial half-sib pedigree scaffold was merged into all larval and adult records before modeling.
(TIF)

S3 Table Composite tolerance scores, cross-stage family association and rank reversal. Stage-specific OA tolerance is represented as a weighted geometric mean of the dominant demographic components for each life stage. Cross-stage ranking is based on these composite scores rather than on a single trait; robustness analyses are summarized in S4–S6 Tables.
(XLSX)

S4 Table Rank reversal, permutation, threshold sweep and leave-one-sire/dam robustness. Permutation tests, top-set reversals and pedigree-group leave-one-out diagnostics support the negative cross-stage association reported in Fig 2.
(XLSX)

S5 Table Robustness of the cross-stage family association to alternative composite definitions. Negative larval-adult association remains evident across equal-weight, arithmetic-ratio, and adult-trait-exclusion variants.
(XLSX)

S6 Table Bivariate pedigree animal model and sire/dam covariance summaries. Model: y = Xβ + a + e, with a ~ N(0, A ⊗ G) and e ~ N(0, I ⊗ R). A is the numerator relationship matrix derived from the partial half-sib pedigree. Because phenotypes are family means on a partial pedigree, this table supports covariance-sign inference rather than serving as the sole primary effect-size estimate.
(XLSX)

S7 Table Pedigree-aware mixed model for larval survival. Empirical-logit vial survival ~ Treatment + retained random intercepts for block, dam, and family.
(XLSX)

S8 Table Pedigree-aware linear mixed model for larval shell area. Shell area ~ Treatment + retained random intercepts for block, dam, and family.
(XLSX)

S9 Table Pedigree-aware linear mixed model for larval shell thickness. Shell thickness ~ Treatment + retained random intercepts for block, dam, and family.
(XLSX)

S10 Table Pedigree-aware linear mixed model for larval shell hardness. Shell hardness ~ Treatment + retained random intercepts for block, dam and family.
(XLSX)

S11 Table Pedigree-aware linear mixed model for metamorphosis timing. Days to metamorphosis ~ Treatment + retained random intercepts for block, dam, and family.
(XLSX)

S12 Table Selection on larval shell area and developmental time. Standardized selection differentials *S*′ for shell area and days to metamorphosis, with the retained quadratic term for days to metamorphosis, plotted in Fig 4e. Each value is an ordinary-least-squares regression of vial-level relative survival on one standardized trait, fitted separately within each treatment, with traits standardized across the pooled data and relative fitness computed within treatment; the multivariate selection gradients *β* used for the predicted responses are reported in S19 Table. Projection outputs are reported separately in S20–S22 Tables.
(XLSX)

S13 Table Pedigree-aware linear mixed model for adult shell growth. Shell growth ~ Treatment + retained random intercepts for dam and family. Candidate sire term screened within the partial half-sib pedigree.
(XLSX)

S14 Table Pedigree-aware mixed model for adult shell dissolution. Dissolution score ~ Treatment + retained random intercepts for dam and family.
(XLSX)

S15 Table Pedigree-aware linear mixed model for adult metabolic rate. MO_2_ ~ Treatment + retained random intercepts for dam and family.
(XLSX)

S16 Table Pedigree-aware linear mixed model for adult gonadal index. Gonadal index after two-year ambient pond grow-out ~ Treatment during the 90-day OA nursery phase + retained random intercepts for dam and family.
(XLSX)

S17 Table Among-family covariance (correlation-scale) matrix G for nine traits, estimated separately within the acidified and the ambient environment. Correlation-scale matrices for the five larval and four post-metamorphic traits described in Materials and methods §7.4, with eigenvalues, effective dimensionality, family-cluster bootstrap intervals and *P* values against the design-based null in which cross-stage covariance is set to zero. Traits were standardized to unit variance before the among-family decomposition.
(XLSX)

S18 TableTrait loadings on the leading eigenvector $g_{max}$ of the among-family covariance matrix.Loadings under acidification and under ambient conditions at the family-level, together with the among-sire (paternal half-sib) loadings used to check that the conflict axis is present in the additive component.(XLSX)

S19 TableSelection geometry on the estimated among-family covariance matrix.Stage-specific standardized selection gradients, the predicted multivariate responses R = Gβ summed within each stage with family-cluster bootstrap intervals, the angles between predicted responses and stage-specific selection gradients, and evolvabilities. The matrix-comparison statistics contrasting the acidified and ambient matrices are given in S17 Table.(XLSX)

S20 Table Family-cluster bootstrap endpoints and paired-difference probabilities. Family-cluster bootstrap endpoints and paired probabilities supplementing Fig 6j–6l.
(XLSX)

S21 Table Continuous stage-weight scan for the empirical conflict model. Generation-20 outcomes were evaluated across a continuous larval-weight gradient (adult weight = 1 − larval weight).
(XLSX)

S22 Table Projection sensitivity to forcing strength and time horizon. Projections for all three anchor weightings were rerun across alternative base selection strengths (*α*_0_), OA forcing ramps (*β*), and generation horizons, using the same model as S20 Table and Fig 6.
(XLSX)

S23 Table Among-family variance components and the eigen-analysis repeated on the correlation scale. Trait-by-trait among-family variance in each environment, with the leading-axis share, effective dimensionality and *g*_max_ loadings recomputed from the correlation matrices of S17 Table, and a cross-substitution of correlation structure and variance scale between the two environments. Because every trait is standardized to unit total variance within its environment, each variance component is that trait’s among-family share and their sum is the overall among-family variation.
(XLSX)

S1 Data Larval vial-level source data. Each row is one vial. FamilyID, Sire, and Dam are merged from the partial half-sib pedigree scaffold so every record can be traced to the mating design and to the source data used for Figs 2, 3, and 4.
(XLSX)

S2 Data Adult individual-level source data. Each row is one post-metamorphic individual. FamilyID, Sire, and Dam are merged from the partial half-sib pedigree scaffold so every record can be traced to the mating design and to the source data used for Figs 2, 5, and 6. Shell growth, dissolution, and MO_2_ derive from the 90-day OA nursery phase; gonadal index was quantified after all juveniles were returned to ambient seawater pond culture for 2 years.
(XLSX)

## Abbeviations

OA: ocean acidification
ICCs: intraclass correlations.

## References

[1] Caldeira K, Wickett ME. Anthropogenic carbon and ocean pH. Nature. 2003;425(6956):365–365. doi: 10.1038/425365a 14508477

[2] Feely RA, Sabine CL, Lee K, Berelson W, Kleypas J, Fabry VJ, et al. Impact of anthropogenic CO_2_ on the CaCO_3_ system in the oceans. Science. 2004;305(5682):362–6. doi: 10.1126/science.1097329 15256664

[3] Orr JC, Fabry VJ, Aumont O, Bopp L, Doney SC, Feely RA, et al. Anthropogenic ocean acidification over the twenty-first century and its impact on calcifying organisms. Nature. 2005;437(7059):681–6. doi: 10.1038/nature04095 16193043

[4] Doney SC, Fabry VJ, Feely RA, Kleypas JA. Ocean acidification: the other CO_2_ problem. Ann Rev Mar Sci. 2009;1:169–92. doi: 10.1146/annurev.marine.010908.163834 21141034

[5] Wilbur HM. Complex life cycles. Annu Rev Ecol Syst. 1980;11:67–93.

[6] Werner EE, Gilliam JF. The ontogenetic niche and species interactions in size-structured populations. Annu Rev Ecol Syst. 1984;15:393–425.

[7] Moran NA. Adaptation and constraint in the complex life cycles of animals. Annu Rev Ecol Syst. 1994;25:573–600.

[8] Marshall DJ, Morgan SG. Ecological and evolutionary consequences of linked life-history stages in the sea. Curr Biol. 2011;21(18):R718-25. doi: 10.1016/j.cub.2011.08.022 21959162

[9] Kroeker KJ, Kordas RL, Crim RN, Singh GG. Meta-analysis reveals negative yet variable effects of ocean acidification on marine organisms. Ecol Lett. 2010;13(11):1419–34. doi: 10.1111/j.1461-0248.2010.01518.x 20958904

[10] Gazeau F, Parker LM, Comeau S, Gattuso JP, O’Connor WA, Martin S. Impacts of ocean acidification on marine shelled molluscs. Mar Biol. 2013;160:2207–45.

[11] Kelly MW, Padilla-Gamiño JL, Hofmann GE. Natural variation and the capacity to adapt to ocean acidification in the keystone sea urchin *Strongylocentrotus purpuratus*. Glob Chang Biol. 2013;19(8):2536–46. doi: 10.1111/gcb.12251 23661315

[12] Kroeker KJ, Kordas RL, Crim R, Hendriks IE, Ramajo L, Singh GS, et al. Impacts of ocean acidification on marine organisms: quantifying sensitivities and interaction with warming. Glob Chang Biol. 2013;19(6):1884–96. doi: 10.1111/gcb.12179 23505245 PMC3664023

[13] Parker LM, Ross PM, O’Connor WA, Pörtner HO, Scanes E, Wright JM. Predicting the response of molluscs to the impact of ocean acidification. Biology (Basel). 2013;2(2):651–92. doi: 10.3390/biology2020651 24832802 PMC3960890

[14] Pespeni MH, Sanford E, Gaylord B, Hill TM, Hosfelt JD, Jaris HK, et al. Evolutionary change during experimental ocean acidification. Proc Natl Acad Sci U S A. 2013;110(17):6937–42. doi: 10.1073/pnas.1220673110 23569232 PMC3637708

[15] Johnson DW. Selection on offspring size and contemporary evolution under ocean acidification. Nat Clim Chang. 2022;12:757–60.

[16] Lande R. Quantitative genetic analysis of multivariate evolution, applied to brain: body size allometry. Evolution. 1979;33(1Part2):402–16. doi: 10.1111/j.1558-5646.1979.tb04694.x 28568194

[17] Lande R, Arnold SJ. The measurement of selection on correlated characters. Evolution. 1983;37(6):1210–26. doi: 10.1111/j.1558-5646.1983.tb00236.x 28556011

[18] Blows MW, Hoffmann AA. A reassessment of genetic limits to evolutionary change. Ecology. 2005;86(6):1371–84. doi: 10.1890/04-1209

[19] Walsh B, Blows MW. Abundant genetic variation strong selection = multivariate genetic constraints: a geometric view of adaptation. Annu Rev Ecol Evol Syst. 2009;40:41–59.

[20] Hine E, McGuigan K, Blows MW. Evolutionary constraints in high-dimensional trait sets. Am Nat. 2014;184(1):119–31. doi: 10.1086/676504 24921605

[21] Byrne M. Impact of ocean warming and ocean acidification on marine invertebrate life history stages: vulnerabilities and potential for persistence in a changing ocean. Oceanogr Mar Biol Annu Rev. 2011;49:1–42.

[22] Pörtner HO, Bock C, Mark FC. Oxygen- and capacity-limited thermal tolerance: bridging ecology and physiology. J Exp Biol. 2017;220:2685–96. 10.1242/jeb.134585 28768746

[23] Fisher RA. The genetical theory of natural selection: a complete variorum edition. Oxford: Oxford University Press; 1999.

[24] Orr HA. Fitness and its role in evolutionary genetics. Nat Rev Genet. 2009;10(8):531–9. doi: 10.1038/nrg2603 19546856 PMC2753274

[25] Tenaillon O. The utility of fisher’s geometric model in evolutionary genetics. Annu Rev Ecol Evol Syst. 2014;45:179–201. doi: 10.1146/annurev-ecolsys-120213-091846 26740803 PMC4699269

[26] Williams GC. Pleiotropy, natural selection, and the evolution of senescence. Evolution. 1957;11(4):398. doi: 10.2307/2406060

[27] Roff DA, Fairbairn DJ. The evolution of trade-offs: where are we? J Evol Biol. 2007;20:433–47. 10.1111/j.1420-9101.2006.01255.x 17305809

[28] Connallon T, Clark AG. A general population genetic framework for antagonistic selection that accounts for demography and recurrent mutation. Genetics. 2012;190(4):1477–89. doi: 10.1534/genetics.111.137117 22298707 PMC3316657

[29] Aguirre JD, Blows MW, Marshall DJ. The genetic covariance between life cycle stages separated by metamorphosis. Proc Biol Sci. 2014;281(1788):20141091. doi: 10.1098/rspb.2014.1091 24966319 PMC4083807

[30] Falconer DS. Introduction to quantitative genetics. London: Longman; 1981.

[31] Richardson EL, Marshall DJ. Mapping the correlations and gaps in studies of complex life histories. Ecol Evol. 2023;13(2):e9809. doi: 10.1002/ece3.9809 36820248 PMC9937794

[32] Marshall DJ, Burgess SC, Connallon T. Global change, life-history complexity and the potential for evolutionary rescue. Evol Appl. 2016;9(9):1189–201. doi: 10.1111/eva.12396 27695526 PMC5039331

[33] Marshall DJ, Connallon T. Carry-over effects and fitness trade-offs in marine life histories: the costs of complexity for adaptation. Evol Appl. 2022;16(2):474–85. doi: 10.1111/eva.13477 36793690 PMC9923492

[34] Melzner F, Gutowska MA, Langenbuch M, Dupont S, Lucassen M, Thorndyke MC, et al. Physiological basis for high CO_2_ tolerance in marine ectothermic animals: pre-adaptation through lifestyle and ontogeny? Biogeosciences. 2009;6(10):2313–31. doi: 10.5194/bg-6-2313-2009

[35] Pandolfi JM, Connolly SR, Marshall DJ, Cohen AL. Projecting coral reef futures under global warming and ocean acidification. Science. 2011;333(6041):418–22. doi: 10.1126/science.1204794 21778392

[36] Arnold SJ, Wade MJ. On the measurement of natural and sexual selection: theory. Evolution. 1984;38(4):709–19. doi: 10.1111/j.1558-5646.1984.tb00344.x 28555816

[37] Sanford E, Kelly MW. Local adaptation in marine invertebrates. Ann Rev Mar Sci. 2011;3:509–35. doi: 10.1146/annurev-marine-120709-142756 21329215

[38] Reusch TBH. Climate change in the oceans: evolutionary versus phenotypically plastic responses of marine animals and plants. Evol Appl. 2014;7(1):104–22. doi: 10.1111/eva.12109 24454551 PMC3894901

[39] Bell G, Gonzalez A. Evolutionary rescue can prevent extinction following environmental change. Ecol Lett. 2009;12(9):942–8. doi: 10.1111/j.1461-0248.2009.01350.x 19659574

[40] Gonzalez A, Ronce O, Ferriere R, Hochberg ME. Evolutionary rescue: an emerging focus at the intersection between ecology and evolution. Philos Trans R Soc Lond B Biol Sci. 2013;368(1610):20120404. doi: 10.1098/rstb.2012.0404 23209175 PMC3538460

[41] Carlson SM, Cunningham CJ, Westley PAH. Evolutionary rescue in a changing world. Trends Ecol Evol. 2014;29(9):521–30. doi: 10.1016/j.tree.2014.06.005 25038023

[42] Bell G. Evolutionary rescue. Annu Rev Ecol Evol Syst. 2017;48(1):605–27. doi: 10.1146/annurev-ecolsys-110316-023011

[43] Wilson AJ, Réale D, Clements MN, Morrissey MM, Postma E, Walling CA, et al. An ecologist’s guide to the animal model. J Anim Ecol. 2010;79(1):13–26. doi: 10.1111/j.1365-2656.2009.01639.x 20409158

[44] Marshall DJ, Krug PJ, Kupriyanova EK, Byrne M, Emlet RB. The biogeography of marine invertebrate life histories. Annu Rev Ecol Evol Syst. 2012;43:97–114.

[45] Sunday JM, Calosi P, Dupont S, Munday PL, Stillman JH, Reusch TBH. Evolution in an acidifying ocean. Trends Ecol Evol. 2014;29(2):117–25. doi: 10.1016/j.tree.2013.11.001 24355315

[46] Gienapp P, Teplitsky C, Alho JS, Mills JA, Merilä J. Climate change and evolution: disentangling environmental and genetic responses. Mol Ecol. 2008;17(1):167–78. doi: 10.1111/j.1365-294X.2007.03413.x 18173499

[47] Hoffmann AA, Sgrò CM. Climate change and evolutionary adaptation. Nature. 2011;470(7335):479–85. doi: 10.1038/nature09670 21350480

[48] Andreassen AH, Clements JC, Morgan R, Spatafora D, Metz M, Åsheim ER, et al. Evolution of warming tolerance alters physiology and life history traits in zebrafish. Nat Clim Chang. 2025;15(6):665–72. doi: 10.1038/s41558-025-02332-y 40520441 PMC12158752

[49] Hofmann GE, Barry JP, Edmunds PJ, Gates RD, Hutchins DA, Klinger T. The effect of ocean acidification on calcifying organisms in marine ecosystems: an organism-to-ecosystem perspective. Annu Rev Ecol Evol Syst. 2010;41:127–47.

[50] Henderson CR. Best linear unbiased estimation and prediction under a selection model. Biometrics. 1975;31(2):423–47. doi: 10.2307/2529430 1174616

[51] Riebesell U, Fabry VJ, Hansson L, Gattuso JP. Guide to best practices for ocean acidification research and data reporting. Luxembourg: Publications Office of the European Union; 2011.

[52] Lewis E, Wallace DWR. Program developed for CO_2_ system calculations. Oak Ridge (TN): Carbon Dioxide Information Analysis Center; 1998.

[53] Dickson AG, Sabine CL, Christian JR. Guide to Best Practices for Ocean CO_2_ Measurements. Sidney (BC): North Pacific Marine Science Organization; 2007.

[54] Bates D, Mächler M, Bolker BM, Walker SC. Fitting linear mixed-effects models using lme4. J Stat Softw. 2015;67:1–48.

[55] Brooks ME, Kristensen K, van Benthem KJ, Magnusson A, Berg CW, Nielsen A. glmmTMB balances speed and flexibility among packages for zero-inflated generalized linear mixed modeling. R J. 2017;9:378–400.

[56] Warton DI, Hui FKC. The arcsine is asinine: the analysis of proportions in ecology. Ecology. 2011;92(1):3–10. doi: 10.1890/10-0340.1 21560670

[57] Efron B, Tibshirani RJ. An introduction to the bootstrap. New York: Chapman & Hall/CRC; 1993.

[58] Patterson HD, Thompson R. Recovery of inter-block information when block sizes are unequal. Biometrika. 1971;58(3):545–54. doi: 10.1093/biomet/58.3.545

[59] Morrissey MB, Hangartner S, Monro K. A note on simulating null distributions for G matrix comparisons. Evolution. 2019;73(12):2512–7. doi: 10.1111/evo.13842 31502676

[60] Hine E, Blows MW. Determining the effective dimensionality of the genetic variance-covariance matrix. Genetics. 2006;173(2):1135–44. doi: 10.1534/genetics.105.054627 16547106 PMC1526539

[61] Krzanowski WJ. Between-groups comparison of principal components. J Am Stat Assoc. 1979;74:703–7.

[62] Chirgwin E, Marshall DJ, Sgrò CM, Monro K. The other 96%: Can neglected sources of fitness variation offer new insights into adaptation to global change? Evol Appl. 2016;10(3):267–75. doi: 10.1111/eva.12447 28250811 PMC5322406

[63] Ewens WJ. Mathematical population genetics I: theoretical introduction. 2nd ed. New York: Springer; 2004.

